# Maintaining Homeostasis by Decision-Making

**DOI:** 10.1371/journal.pcbi.1004301

**Published:** 2015-05-29

**Authors:** Christoph W. Korn, Dominik R. Bach

**Affiliations:** 1 Department of Psychiatry, Psychotherapy, and Psychosomatics, University of Zurich, Zurich, Switzerland; 2 Wellcome Trust Centre for Neuroimaging, University College London, London, United Kingdom; Oxford University, UNITED KINGDOM

## Abstract

Living organisms need to maintain energetic homeostasis. For many species, this implies taking actions with delayed consequences. For example, humans may have to decide between foraging for high-calorie but hard-to-get, and low-calorie but easy-to-get food, under threat of starvation. Homeostatic principles prescribe decisions that maximize the probability of sustaining appropriate energy levels across the entire foraging trajectory. Here, predictions from biological principles contrast with predictions from economic decision-making models based on maximizing the utility of the endpoint outcome of a choice. To empirically arbitrate between the predictions of biological and economic models for individual human decision-making, we devised a virtual foraging task in which players chose repeatedly between two foraging environments, lost energy by the passage of time, and gained energy probabilistically according to the statistics of the environment they chose. Reaching zero energy was framed as starvation. We used the mathematics of random walks to derive endpoint outcome distributions of the choices. This also furnished equivalent lotteries, presented in a purely economic, casino-like frame, in which starvation corresponded to winning nothing. Bayesian model comparison showed that—in both the foraging and the casino frames—participants’ choices depended jointly on the probability of starvation and the expected endpoint value of the outcome, but could not be explained by economic models based on combinations of statistical moments or on rank-dependent utility. This implies that under precisely defined constraints biological principles are better suited to explain human decision-making than economic models based on endpoint utility maximization.

## Author Summary

Common decision-making models arise from firm axiomatic foundations but do not account for a variety of empirically observed choice patterns such as risk attitudes in the face of high-impact events. Here, we argue that one reason for this mismatch between theory and data lies in the neglect of basic biological principles such as metabolic homeostasis. We use Bayesian model comparison to show that models based on homeostatic considerations explain human decisions better than classic economic models—both in a novel virtual foraging task and in standard economic gambles. Specifically, we show that in line with the principle of homeostasis human choice minimizes the probability of reaching a lower bound. Our results highlight that predictions from biological principles provide simple, testable, and ecologically rational explanations for apparent biases in decision-making.

## Introduction

Homeostasis is paramount to all living organisms [1]. Put simply, organisms have to maintain their internal milieu within certain boundaries to avoid dying. This homeostatic principle reverberates on the levels of molecular interactions [2], hormonal feedback loops [3,4], neural circuits [5], and psychophysiological processes [6]. Beyond the need for immediate regulation, many species face complex decisions with delayed and probabilistic consequences for long-term metabolic homeostasis. Here, we hypothesize that homeostatic requirements guide foraging decisions in humans. For example, hunting deer provides a large energy gain with low probability of obtaining it, while collecting berries provides a small energy gain with high probability. In order to minimize the probability of starvation, human agents should integrate the statistics of the available options with their current energy levels and with their time horizon.

Classical views of homeostasis [1–7] are often illustrated with a thermostat that senses the difference between a temperature set point and the current temperature. This deviation value elicits a change in heating levels. The thermostat is thus supposed to retrospectively compensate deviations that have already manifested themselves. In contrast, we propose that decision-makers can anticipate possible deviations and proactively minimize the probability of reaching a prohibitive boundary such as starvation. This extends established notions of homeostasis in (psycho)physiology [1–7] and is concordant with recent theoretical accounts of homeostasis as a principle explaining decision-making in healthy and psychiatric populations [8–10].

When applied to individual decision-making, predictions from this model are in contradistinction to economic models which firmly rest upon axiomatic foundations [11] and elegantly explain many types of monetary decisions [12]. These models posit that decision-makers base their choices on the utility assigned to the endpoint outcome of a choice irrespective of the trajectory to this endpoint [12–14].

In risk-return models and their variants [13,15], the endpoint utility is computed via statistical moments of an outcome distribution, usually expected value, variance, and in some models also skewness. A considerable literature has generalized risk-return models to include subjective transformations of statistical moments [15,16]. In behavioral economics, variants of risk-return models have been widely used to describe how humans choose between monetary gambles [17–19], how they assess real-life events [16], and how animals decide on primary reinforcers [20,21]. Expected utility theory and its derivations constitute another class of models in which values of possible outcomes are transformed into an internal utility measure by a decision-maker's individual utility function [12,15,22]. Rank-dependent utility models additionally supplement a non-linear weighting of the option’s outcome probabilities [23,24]. Similar to risk-return models, rank-dependent utility models have been used extensively to describe empirical data both from the lab and the field [15,23,24].

Empirically observed deviations from predictions of these microeconomic models are often framed as irrational biases, and additional parameters are included to absorb such biases, but often without making principled assumptions on why these influences should arise in the first place [25]. Here, we furnish a principled biological reason for deviations from economic principles. Critically, maximizing the utility of the endpoint outcome of a given set of options neglects the catastrophic consequences if the trajectory to this outcome reaches a lower bound of the internal milieu. We sought to show that even in a safe laboratory environment, a decision-making model based on homeostatic principles could explain foraging decisions better than economic models. Further, we hypothesized that homeostatic considerations would also guide human decision-making for simply structured lotteries without any reference to foraging, as often employed in behavioral economics [12–14,26].

**Fig 1:**
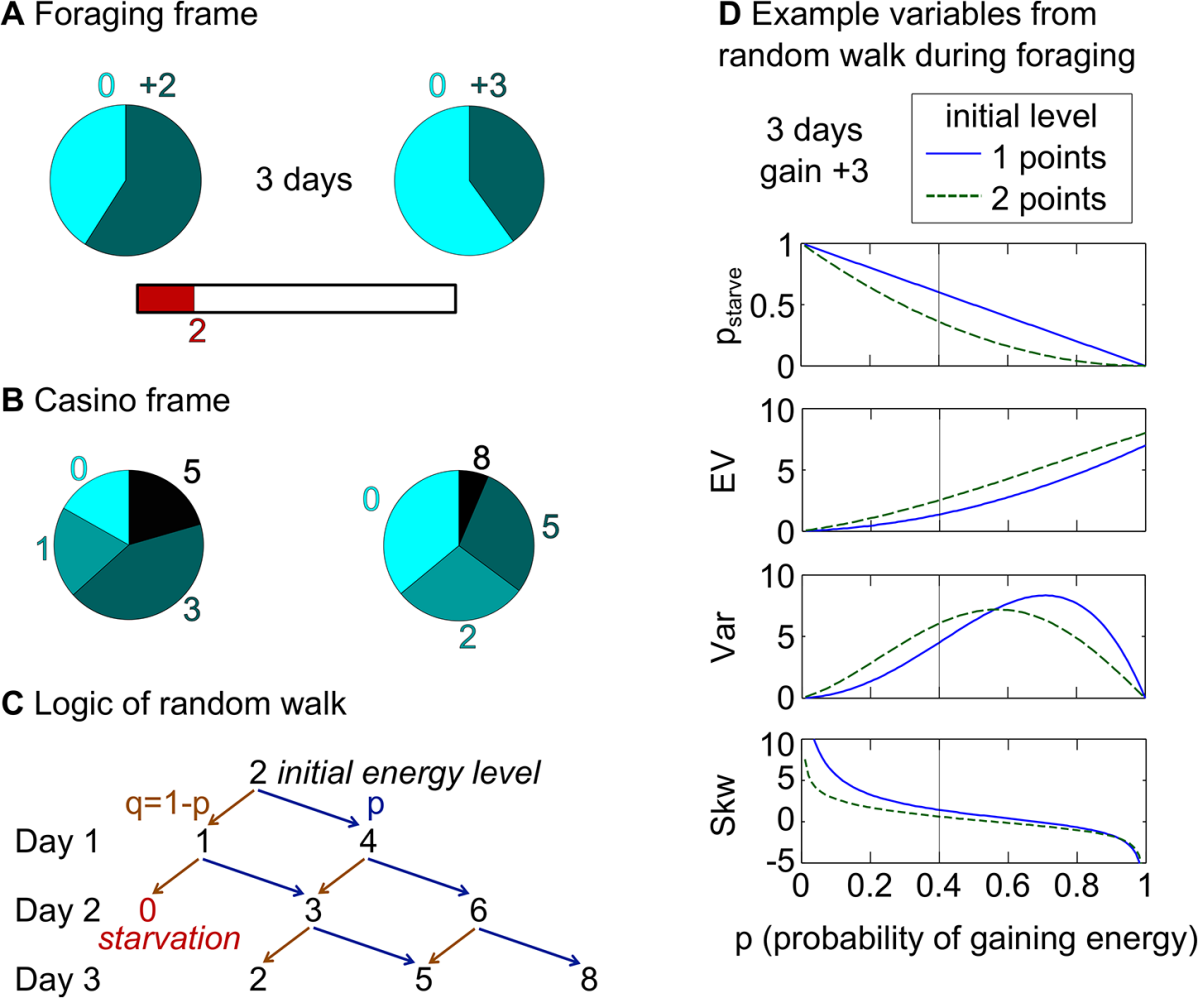
Virtual foraging task and derivation of the gambles. **(A)** Foraging frame: In each trial, participants saw an energy bar depicting their initial energy points. Participants had to decide between two foraging options, which were depicted as pie charts with two sectors: the light blue sectors corresponded to unsuccessful foraging (i.e., to a gain of zero points), the dark blue sectors corresponded to successful foraging (i.e., to a gain of the number of points written above the sectors). In each trial, participants made a single decision and the chosen foraging option was played out for the specified number of “days.” For each day, the probabilistic outcome of the chosen foraging option was determined and the respective gains were added to the energy bar. A sure cost of one point was deducted on each day to mirror energy consumption. If at any day the energy bar reached zero, the participant died from starvation in that trial. We denote this probability as p_starve_. The choice in the foraging frame was therefore between two gamble sequences, where the number of days indicated the number of gambles in each sequence. Participants did not see the outcomes of their choices but were presented with examples in the written instructions. **(B)** Casino frame: Numerically identical gambles as in the foraging frame were presented as pie charts similar to wheel-spinning gambles in a casino. The size of each sector denoted the probability of winning the amount written next to it. In the casino frame, p_starve_ was directly visible as the size of the sector for an outcome of zero. The choice in the casino frame was thus between two single-step gambles. **(C)** Illustration of the logic behind the mathematics of random walks, which we used to derive the gambles (see S1 Text for mathematical details). The tree-like illustration depicts the random walk used to calculate the variables of the right gambles in A and B. The random walk starts at position “2” which corresponds to the initial number of energy points. In each step (“day”) the agent walks to the “right” with probability p (corresponding to successful foraging; dark blue sector of pie chart in A) or to the “left” with probability 1-p (corresponding to unsuccessful foraging; light blue sector of pie chart in A). The step size to the “left” corresponds to the sure cost and is always one. The step size to the right depends on the amount of energy points to gain (here there are +3 points and thus the step size is +2 because the sure cost has to be subtracted). Zero is an absorbing boundary and thus no arrows start from zero. The possible outcomes are directly visible in the casino frame (here 0, 2, 5, and 8 points). To determine the probability of a specific outcome one has to follow all possible paths along the branches of the tree leading to that outcome and sum over their probabilities. The probabilities of a specific “path along the branches” are determined by multiplying the probabilities of all arrows on that path. In the current example, the probability of an outcome of zero (i.e., p_starve_) is q^2^ and the probability of an outcome of 2 is 2pq^2^. **(D)** Distribution of the variables for the random walk shown in C across all values of p (i.e., the probability of successful foraging or of going right in the random walk). In the example of the right gamble shown in A and B, p was chosen at 40% and the intersection of the black vertical lines with the green lines give the variables for the current example (i.e., for an initial energy level of 2 energy points).

To test these hypotheses, we developed a virtual foraging task. In each trial, human participants chose between two “foraging environments” with different possible “energy” gains and associated probabilities, in which they would forage for up to three consecutive “days” (see Fig 1A for illustration). At the time of choice, an energy bar depicted participants’ current internal state. Participants were instructed that on each foraging day, they would lose one energy point, and gain energy according to the statistics of the chosen environment. Successive days in the task required the integration of risks from multiple foraging attempts. For each trial, participants made a single decision between the two foraging environments for the indicated number of days. Losing all energy points on any day in a given foraging period was framed as “starvation” but was not explicitly punished. Each trial was independent. We did not give feedback on choice outcomes of their choices or intermediate states of the foraging sequence. At the end of the experiment, participants were rewarded for the endpoint foraging outcome of two randomly selected trials. Starvation meant that participants did not win anything from the trial. We hypothesized that participants would compute the probability of starvation for the foraging environments and base their decisions on this metric.

We used the mathematics of random walks to analytically derive the distributions of the endpoint outcomes of the foraging period, and of the probabilities of starvation during foraging (see Fig 1C for a graphical illustration, Fig 1D for an example of the variables derived by this procedure, Table 1 for a summary of the gambles, and Methods and S1 Text for mathematical details). Because participants were only rewarded for endpoint outcomes, homeostatic principles are irrelevant for maximizing utility, yet our task was suggestive of using them. Hence, we tested whether such principles also influence decisions when they are not invoked by the task frame. We presented participants with purely economic gambles, framed as wheel-spinning casino lotteries without any reference to foraging (see Fig 1B). These lotteries had identical endpoint outcomes as the foraging environments. The probability of starvation in the foraging frame corresponded to the probability of winning nothing in the casino frame. As in the foraging frame, participants did not receive feedback on the outcomes of their choices. In addition to the instruction, the two frames differed in the fact that options in the foraging frame were presented as gamble sequences when the number of days was greater than one, whereas options in the casino frame were always single-step gambles. To avoid foraging instructions influencing behavior in the casino frame, the casino frame preceded the foraging frame for all participants.

**Table 1:** Variables of the 480 binary gambles used in the task.

Variable	Range	Mean	SD
Initial state (x_0_)	1–2	-	-
Days (n)	1–3	-	-
Gains (g)	2–6	-	-
Probabilities of individual options	0.008–0.9	-	-
EV	1.47–5.53	3.18	1.16
Var	0.75–27.59	8.45	6.61
Skw	-2.67–2.00	0.00	0.76
p_starve_	0.06–0.77	0.35	0.16

The means and SD of EV, Var, Skw, and p_starve_ were calculated over both options of the binary gambles. SD, standard deviation; EV, expected value; Var, variance; Skw, skewness; p_starve_ starvation probability

## Results

### Overall comparison of model families across both frames

We first asked whether models based on homeostatic principles explain choice better than standard economic models—both in a foraging and in a casino frame. We combined choices from both frames and compared three families of formal decision-making models. The first two model families included variations of two types of economic models while the third family comprised models based on homeostatic considerations (see **Methods** and Table 2 for details).

**Table 2:** Model family comparison: Relative log-group Bayes factors.

	Relative log-group Bayes factors (smaller is better)								
	Family 1				Family 2		Family 3		
	Moments without p_starve_				Rank-dependent utility		Moments and p_starve_		
	Model	Model	Model	Model	Model	Model	Model	Model	Model
	1	2	3	4	5	6	7	8	9
	EV	EV	EV	EV	Prelec-I	Prelec-II	EV	EV	EV
		Var	Skw	Var			p_starve_	Var	Var
				Skw				p_starve_	Skw
									p_starve_
All	0	-1333	-1984	-2032	-2154	-2148	**-2191**	-2167	-2126
Foraging	0	-884	-1390	-1437	-1466	-1390	-1511	-1532	**-1569**
Casino	0	-709	-945	-967	-1040	-1023	**-1062**	-1044	-970
Foraging-block 1	0	-472	-683	-696	-734	-679	**-781**	-751	-741
Foraging-block 2	0	-412	-661	-729	-725	-689	-751	-737	**-773**
Casino-block 1	0	-363	-451	-456	-488	-465	**-518**	-487	-439
Casino-block 2	0	-342	-519	-507	-525	-463	**-565**	-537	-487

For a fixed-effects analysis, log-group Bayes factors based on BIC were calculated relative to the simplest model (Model 1). Smaller log-group Bayes factors indicate more evidence for the respective model versus the baseline model. The log-group Bayes factors of the winning models according to fixed-effects analyses are written in bold font. The models included free parameters for the respective variables listed. BIC, Bayesian information criterion; EV, expected value; Var, variance; Skw, skewness; p_starve_ starvation probability

**Fig 2:**
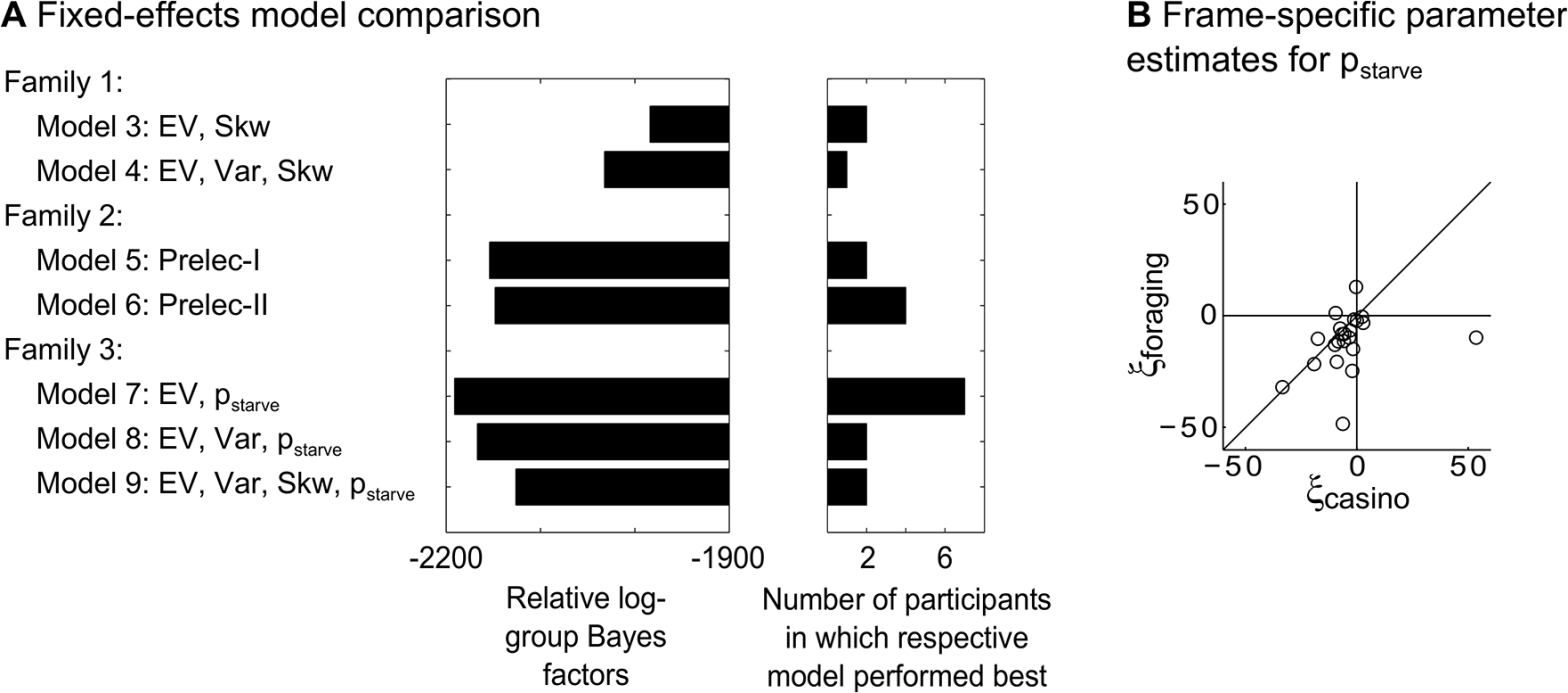
Results of model comparison. **(A)** Log-group Bayes factors (smaller is better) relative to the simplest models (Model 1) based on BIC for the nine models tested (*left part*) and histogram of best-performing models per participant (*right part*). The models belonged to three families. In the first family, models were based on various combinations of weighting parameters for the first three statistical moments (i.e., EV, Var, and Skw). The second family comprised two rank-dependent utility models in which probabilities were weighted according to non-linear weighting functions (Prelec-I and Prelec-II). In the third family, models were based on the homeostatic principle of minimizing p_starve_ in addition to combinations of weighting parameters for the statistical moments. Across both frames, the third model family provided the best fit to the data. For ease of reference, the first two models are not depicted because their log-group Bayes factors were far off relative to the other models. The histogram shows that in 11 of 22 participants the best performing model belonged to the third model family. Note that the fixed-effects analyses do not account for possible outliers, while random-effects analyses do. Smaller log-group Bayes factors indicate more evidence for the respective model versus the baseline model. See also Table 2. **(B)** Parameter estimates from an adaptation of the overall winning model for foraging-p_starve_ (ξ_foraging_) and casino-p_starve_ (ξ_casino_) for individual participants. As expected from the notion that participants should minimize p_starve_, most parameter estimates were negative (i.e., in the lower left quadrant). Additionally, for most participants foraging-p_starve_ was more negative than casino-p_starve_ (i.e., most points lie below the identity line). See also S1 Fig for binned choice data.

In line with our hypothesis that participants’ decisions should take into account the probability of starvation (p_starve_), the homeostatic model family provided the significantly best fit. Under the assumption that different participants may use different models (random-effects analysis), the exceedance probability that the homeostatic model family is the most frequent in the population was 0.9403 (Table 3). Under the assumption that all participants use the same model (fixed-effects analysis), the winning model belonged to the homeostatic family (see Fig 2A and Table 2 for log-group Bayes factors based on the Bayesian information criterion (BIC) relative to the simplest model; see S2 Text Section 7 and S1 Table and S2 Table for results based on the Akaike information criterion, AIC; see S3 Table for the fits of the different models for each individual participant). Thus, the overall comparison of model families confirmed our hypothesis that starvation probability and thus homeostatic principles provided explanatory power in explaining participants’ behavior, over and above economic variables, and although irrelevant for utility maximization in the laboratory.

**Table 3:** Model family comparison: Exceedance probabilities.

	Exceedance probabilities (higher is better)		
	Family 1	Family 2	Family 3
	Moments without p_starve_	Rank-dependent utility	Moments and p_starve_
All	0.0068	0.0529	**0.9403**
Foraging	0.0127	0.0004	**0.9869**
Casino	0.0367	0.0958	**0.8675**
Foraging-block 1	0.0021	0.0000	**0.9979**
Foraging-block 2	0.2804	0.0096	**0.7100**
Casino-block 1	0.0116	0.0197	**0.9687**
Casino-block 2	0.0673	0.0043	**0.9284**

The highest exceedance probabilities according to random-effects analyses are written in bold font. BIC, Bayesian information criterion; p_starve_ starvation probability

### Frame-specific comparison of model families

Next, we separately analyzed choices in the foraging frame and in the purely monetary context of the casino frame, by comparing the three model families within each frame. The same overall pattern emerged. The homeostatic family, in which models included p_starve_, had the highest exceedance probabilities in both frames independently (foraging: 0.9869; casino: 0.8675; Table 3). Also, the models winning in fixed-effects analyses belonged to the homeostatic family (see Table 2 for log-group Bayes factors based on BIC). Further, when we fitted the models separately for the first and second blocks of the foraging and casino frames, the same pattern emerged in all analyses. The homeostatic family had the highest exceedance probabilities and models belonging to this family won the fixed effects analyses (see Tables 2 and 3; see S2 Text Section 7, S1 Table and S2 Table for results based on AIC).

### Comparison within winning model family

Within the winning model family, we analyzed which specific model best explained choices. In a random-effects analysis, the exceedance probability of the simplest homeostatic model was 0.9786 across both frames (Table 4). Similar results emerged when fitting the models separately within the two frames or separately to the first and second blocks (Table 4; see S2 Text Section 7 and S4 Table for results based on AIC; see S1 Fig for binned choice data). In this winning model, the decision variable was a linear combination of difference in starvation probability (p_starve_) weighed by a parameter ξ, and difference in expected value (EV). This decision variable was transformed into a decision probability by a sigmoid function with another parameter, β.

**Table 4:** Comparison within the winning model family: Exceedance probabilities.

	Exceedance probabilities (higher is better)		
	Family 3		
	Moments and p_starve_		
	Model	Model	Model
	7	8	9
	EV	EV	EV
	p_starve_	Var	Var
		p_starve_	Skw
			p_starve_
All	**0.9780**	0.0009	0.0211
Foraging	**0.8627**	0.0108	0.1265
Casino	**0.9992**	0.0008	0.0000
Foraging-block 1	**0.9882**	0.0000	0.0118
Foraging-block 2	**0.7063**	0.0003	0.2934
Casino-block 1	**1.0000**	0.0000	0.0000
Casino-block 2	**0.9999**	0.0001	0.0000

The highest exceedance probabilities according to random-effects analyses are written in bold font. BIC, Bayesian information criterion; EV, expected value; Var, variance; Skw, skewness; p_starve_ starvation probability

### Comparison of parameters in an adapted version of the winning model

The previous analyses showed that participants consistently used models based on homeostatic principles in both frames. This leaves open the question how participants used p_starve_ and whether this differed between the two frames. Hence, we added frame-specific free parameters to the winning model and then tested the parameter estimates across participants.

Specifically, we adapted the winning model (Model 7), which included two free parameters: a parameter β for the decision noise and a parameter ξ to quantify the impact of p_starve_ on participants’ decision. This parameter ξ was replaced by two frame-specific weighting parameters (ξ_foraging_ and ξ_casino_). We added this model (Model 10) to the initial set of three models in the third family. Despite being penalized for the additional free parameter, it explained choices better than the other three models considered. Its exceedance probability was 0.9983 in a random-effects analysis and it had the smallest log-group Bayes factor in a fixed-effects analysis (Table 5; see S2 Text Section 7 and S5 Table for results based on AIC). This indicates that participants weighted p_starve_ differently in the two frames.

**Table 5:** Comparison of an additional model including frame-specific parameters: Relative log-group Bayes factors and exceedance probabilities.

	Family 3			Additional model
	Moments and p_starve_			
	Model	Model	Model	Model
	7	8	9	10
	EV	EV	EV	EV
	p_starve_	Var	Var	forage-p_starve_
		p_starve_	Skw	casino-p_starve_
			p_starve_	
Relative log-group Bayes factors—all data (smaller is better)	0	23	64	**-797**
Exceedance probabilities—all data (higher is better)	0.0002	0.0001	0.0014	**0.9983**

Log-group Bayes factors based on BIC were calculated relative to the simplest model (Model 7). Smaller log-group Bayes factors indicate more evidence for the respective model versus the baseline model. The log-group Bayes factor of the winning model according to fixed-effects analysis and the highest exceedance probability according to random-effects analysis are written in bold font. BIC, Bayesian information criterion; EV, expected value; Var, variance; Skw, skewness; p_starve_ starvation probability

Crucially, our prediction that participants’ choices minimize p_starve_ requires that weighting parameters of p_starve_ be negative. Indeed, the parameters for the frame-specific parameters ξ_foraging_ and ξ_casino_ were significantly smaller than zero across participants (sign test on parameters in the overall winning model: ξ_foraging_: p<.001; and ξ_casino_: p<.005; Fig 2B). That is, participants chose the gambles with the smaller p_starve_ and thus minimized p_starve_. Additionally, across participants ξ_foraging_ was smaller than ξ_casino_ (sign test comparing ξ_foraging_ and ξ_casino_: p<.05).

In line with the above analyses, supporting analyses showed that p_starve_ played a greater role than EV in the foraging frame, while in the casino frame EV played a greater role than p_starve_, for explaining choices (see S2 Text Section 1 and S6 Table for details). Additionally, we devised a supplemental model to test whether the different number of foraging days led to a different weighting of p_starve_ but did not find evidence supporting this idea (S2 Text Section 2 and S7 Table). We also found no evidence supporting the hypothesis that different combinations of energy levels and foraging days lead to differential weighting of p_starve_ (S2 Text Section 4). Supplementary analysis showed that participants did not erroneously include values below zero in their estimation of the outcome distributions in the foraging frame (S2 Text Section 5 and S8 Table). In an exploratory analysis, we also found no evidence for a relationship of the model parameters to participants’ meta-cognitive risk assessments on the domain-specific risk-attitude scale (S2 Text Section 8).

Taken together, participants consistently minimized p_starve_ in both frames and did so more in the foraging than in the casino frame.

### Analyses of reaction times

Can reaction times (RTs) as a tentative measure of choice difficulty give us additional evidence for the relevance of homeostatic principles? Since our model comparison showed that EV and p_starve_ explained participants’ choices, we tested whether EV and p_starve_ also related to RTs. That is, we tested whether RTs were faster for larger absolute differences between the two options in EV and p_starve_. This was indeed the case as shown by a linear mixed effects model on log-transformed RTs (EV: t = -4. 98, p<.001; p_starve_: t = -2.62, p<.05; significance levels were determined by log-likelihood tests, comparing the full model to a model without the respective factor). The interaction of EV and p_starve_ was significant and related to slower RTs (t = 3.52, p<.005). See S1 Fig for binned RT data. In sum, a combination of EV and p_starve_ was related to choice difficulty as indexed by RTs, which corroborates that homeostatic principles guided participants’ choices.

## Discussion

This study addressed whether homeostatic principles explain human decision-making over and above previously described economic models based on endpoint utility maximization. We found that human decisions minimized the probability of reaching a lower homeostatic bound on the trajectory to their endpoint outcomes, despite the fact that our tasks did not entail any explicit negative consequences of reaching this boundary. This was evident both in a virtual foraging task, in which the possibility of starvation was a salient task feature, and in a casino-like frame, in which only the endpoint outcomes of the gambles and their associated probabilities were explicitly stated. Our fine grained model comparison provided evidence that the decision variable in the most parsimonious model was based on a linear combination of the probability of starvation and endpoint expected value (EV), outperforming standard economic models.

The maximization of endpoint EV lies at the core of many variants of axiomatically derived microeconomic models. However, neither variants of risk-return models nor variants of expected utility theory predict that decisions minimize the probability of reaching a lower homeostatic bound before that endpoint is realized. The winning model family included the probability of zero outcomes although we did incentivize participants to avoid them, and although zero outcomes are already incorporated into the calculation of statistical moments and utilities. For a description of behavior, we could have used a very specific shape of the utility function which assigns a high negative utility to the zero outcome and positive utility to neighboring positive outcome, in contrast to typical utility functions in the economic literature. However, such a model would neither be more parsimonious than ours, nor offer any additional explanatory power.

We note that in the best fitting model, the decision variable was a linear mixture of outcome variables and thus it did not differ from previous risk-return models in its mathematical structure. Crucially, minimization of the probability of a zero outcome provided more explanatory power than risk-attitudes based on variance or skewness. Thus, our results are in line with previous accounts calling for more fine-grained and possibly context-dependent metrics within the framework of risk-return models [19,27]. Additionally, our model only makes meaningful predictions when the probability of threats to homeostasis is nonzero and thus our approach has the desirable feature that the scope of the model is under precisely defined constraints.

We provide evidence that the homeostatic principle of avoiding a lower boundary on energy levels pervades human decision-making. Classical descriptions often relate homeostatic processes to the actions of a thermostat [6,7]. The thermostat example best fits to physiological variables with a narrow homeostatic range, for which this range can be approximated by a set point (e.g., blood pH) [7]. For other variables the homeostatic range is larger. In the case of metabolic homeostasis, glycogen and fat buffers enlarge the homeostatic range and relevant homeostatic counter-measures occur at the boundaries of this range [7]. For simplicity, we assumed starvation to be a hard boundary but the same principle would apply for soft boundaries. More importantly, our results extend the notion exemplified by the thermostat analogy. In line with recent theoretical views on homeostasis in healthy and psychiatric populations [8,10], we conjectured that human decision-makers can estimate the probability of future disruptions to homeostasis. Thus, in contrast to a thermostat that can only react to homeostatic threats once they have occurred, human—and possibly many animal—decision-makers can proactively avoid threats to homeostasis.

The same model performed best in both the foraging frame and in the casino frame. We highlight this similarity between the two frames because it shows that the homeostatic principle of minimizing the probability of a zero outcome is at play even when participants are not primed by the task description to do so. Furthermore, the same model explains behavior in gamble sequences and in single-step gambles. In the casino frame, the probability of starvation was directly depicted by the size of the sector in the pie chart that indicated the probability for the zero outcome. Strikingly, in the foraging frame participants integrated the probabilities of gaining energy over the indicated number of days to compute the probability of starvation. Participants could not learn the outcome distributions through experience because we did not provide them with feedback. Thus, decisions in the foraging frame were not dependent on participants having directly experienced sequences in the virtual foraging environments. Risky decision-making differs depending on whether outcome distributions are described or learned from experience [22,28]. For example, rare events tend to exert less impact in decisions based on experience. Our results suggest that such an underweighting might not occur for the probability of starvation [28].

Within the winning model, more fine grained analyses revealed differences between the two frames in the best-fitting parameter estimates. The probability of starvation in the foraging frame had a greater impact on participants’ decisions than the corresponding probability of receiving nothing in the casino frame—an effect unrelated to the sequential versus single-step presentation of the gambles (see S2 Text Section 3). This was the case even though participants had to compute the probability of starvation in the foraging frame by combining information about internal state, foraging options, and time horizon. Approximating starvation probabilities may become more difficult and thus imprecise as the number of steps increases. In the current study, participants were able to approximate the probability of starvation with sufficient accuracy for at least three steps, as their decisions were based on this metric.

The structure of our tasks complies with the requirements of economic paradigms such as complete knowledge and incentive-compatibility [12]. Thus, specific task characteristics are unlikely to explain why our homeostatic model outperformed standard economic models, based on statistical moments [15,17,18] or non-linear probability weighting [23,24]. Instead, we reason that the biological constraints relevant in ecological contexts such as hunting or farming exert a prevailing impact on human decisions in the laboratory—even if apparently irrelevant to the task at hand. A similar rationale has recently been advocated in discussions of whether animal [29,30] and human [31–34] decision-making deviates from normative models. According to probabilistic accounts of brain function, the brain uses prior probabilities to perform probabilistic inferences [8,14,35,36]. These prior probabilities are tuned—by evolution and/or experience—to the natural statistics of real world environments [30,31]. Consequently, human and non-human decision-makers may behave rationally according to their beliefs but they appear irrational because those beliefs are not warranted in deliberately simplified laboratory tasks or in some other contexts [30,37]. Overall, this recent approach argues for complementing considerations about economic rationality (i.e., maximizing financial gain given currently available information) with considerations about ecological rationality (i.e., maximizing fitness given priors on environmental statistics). Its promise lies in unifying and explaining a diverse set of seemingly irrational behaviors while its challenge lies in identifying and testing the ecological principles on which to base such explanations [30].

The current study demonstrates that a basic biological principle about the internal milieu provides a refined and parsimonious explanation of human decisions under risk. Our virtual foraging task was specifically designed to test the influence of homeostatic principles on risky choice. It thereby relates to, and extends, previous tests of risk-sensitive foraging theory in animals [38–41]. Risk-sensitive foraging theory provides an account of how animal should choose between risky foraging options so as to maximize their fitness [40]. The crucial insight of risk-sensitive foraging theory is that foraging animals should choose options with higher variance if options with lower variance cannot provide a sufficient amount of energy to meet critical levels until a certain time point. For example, hungry birds in winter should become more risk-prone as nightfall approaches. Thus, risk-sensitive foraging theory provides an ecologically rational benchmark [38–40] although empirical evidence for it has been mixed [40]. Similar to risk-sensitive foraging theory, our model comprises a hard boundary that is relevant to the decision-maker within a given time horizon. Crucially, we introduce a novel and simple mathematical description for deriving sequential gambles that mirror foraging settings. Testing the model in a virtual setting in humans circumvents challenges of non-human animal research such as the need to impose actual threats onto participants or the need to impart outcome distributions through extensive training.

Risk-sensitive foraging theory has been related to loss aversion [40], which refers the empirical observation that humans seem to care more about losses than gains of equivalent magnitude [40,42]. One may speculate that loss aversion may be related to our finding that participants minimized the probability of starvation. However, loss aversion can only arise in mixed gambles (i.e., when options entail gains and losses) [23,42], and our gambles did not involve losses. Therefore, loss aversion cannot explain our findings.

Our approach is in line with some recent studies that have employed virtual foraging-like tasks to probe the psychological and neural mechanisms of complex decision-making in animals [41,43] and humans [44–46]. One notable study showed that humans adjust their risk-taking behavior dynamically over a sequence of gambles [44]. Another study provides evidence that humans continuously reassess the sequences of gambles available to them in the future although economically optimal strategies prescribe that decisions be independent of sequence order [47]. Our results complement these findings by suggesting that such behavior could be easily explained if people take into account the probability of “starvation” during the choice sequence. Overall, the current study makes detailed predictions for apparent irrationalities in dynamic foraging tasks that are consistent with earlier reports.

Our model of homeostatic decision-making lends itself to possible extensions. First, decision-makers usually have to maintain several variables in a homeostatic range. Our model can easily be extended to such situations with the prediction that decision-makers minimize the joint probability of starvation, which may imply giving up a large amount of one variable to avoid getting zero of another. When boundaries are soft rather than hard, this can be thought of as minimizing a constrained functional that describes a trajectory through homeostatic space. Second, risk preferences are often assumed to be rather stable personality traits [48] but our model implies that they should vary depending on threats to homeostasis [38]. Third, insurances for rare high-impact events have been a recent focus in economics [49]. The concept of starvation in our model may give a handle on investigating the impact of such events on human decisions.

Our results suggest that the pursuit of this fundamental biological goal translates into simple but specific predictions for decision-making that are amenable to empirical tests. Standard economic models provide an indispensable benchmark against which to test the inclusion of additional considerations about biological considerations [12,15,23]. Commonly, models of risky decision-making have to strike a balance between the elegance of axiomatic economic foundations that are at odds with empirical observations and the unwieldy ad hoc assumptions of irrational biases. Our results provide an example that models based on fundamental biological principles such as homeostasis can reconcile parsimony with an explanation for apparent irrationalities.

## Methods

### Ethics statement

The study was conducted in accord with the Declaration of Helsinki and approved by the governmental research ethics committee (Kantonale Ethikkommission Zürich, KEK-ZH-Nr. 2013–0328). All participants gave written informed consent using a form approved by the ethics committee.

### Participants

Twenty-two participants (15 female; age: mean = 25 years, SD = 5.0) were recruited from a student population via mailing lists of local universities. Participants were paid a show-up fee of CHF 15 plus a variable amount (see below).

### Task

Participants completed 960 trials in two variants (frames) of a binary choice task: the foraging and the casino frames (Fig 1A and 1B). The same list of 480 combinations of gambles was used for both frames (i.e., the outcome distributions were numerically equal; see Table 1 for an overview of the variables; see below and S1 Text for details on how gambles were derived). For both frames, participants received detailed written instructions and performed eight training trials followed by two blocks of the actual task. The task was presented using the MATLAB toolbox Cogent 2000 (www.vislab.ucl.ac.uk). The instruction for the foraging frame told participants to imagine themselves in a hunter-gatherer context. Since we wanted to exclude that putting participants into a foraging mindset influenced choices in the casino frame, all participants completed the casino frame before the foraging frame. The 480 gamble combinations for each frame were split into two blocks, which were counterbalanced for order. Each list contained 80 unique gamble combinations; the remaining 400 gamble combinations were included in both lists. During the game participants did not see the outcomes of their choices. That is, participants were given examples of possible outcomes in the written instruction but they did not directly experience them. At the end of the experiment, one trial from each of the four blocks was randomly chosen. The outcomes of these trials were determined based on participants’ choices and the corresponding amount was paid out (1 point was worth CHF 0.75). Thus, both frames were incentivized in the same way. See Fig 1 and S2 Text for further details.

### Gambles

We used the mathematics of random walks to derive outcome distributions for the 480 combinations of gambles (Table 1). We briefly introduce the basic logic (Fig 1C **)**. For details see S1 Text. In a random walk an imaginary agent starts at a given position on a line of positive integers. The starting position corresponds to the initial number of energy points. The agent makes a number of steps on that line, which correspond to the number of days. In each step, the agent moves “right” with a certain probability p and “left” with the probability q = 1-p. Moving left corresponds to unsuccessful foraging and the step sizes correspond to a fixed cost of one energy point. Moving right corresponds to successful foraging and the step sizes correspond to the variable points to gain (minus the cost of one point). Zero represents an absorbing boundary (i.e., if the agent reaches zero, the random walk stops). The possible positions on the number line after a certain number of steps correspond to the range of outcomes. To obtain the probability of an outcome, all the probabilities of all “branches on the tree” toward that outcome have to be summed up. (The number of “branches” is calculated with a binomial coefficient.) Along a given branch the probabilities (i.e., p or q) have to be multiplied. We created different gambles by varying combinations of starting positions, probabilities of moving right and step sizes to the right. In the current study, we included gambles with four different combinations of starting positions and number of steps (a) starting position 1 and 1 step, (b) starting position 1 and 2 steps, (c) starting position 2 and 2 steps, and (d) starting position 2 and 3 steps (with each combination occurring in 120 combinations of gambles). We used the outcomes and their respective probabilities to calculate the statistical moments of the chosen gambles. The probabilities of reaching zero are denoted p_starve_. Note that in the gambles included in the current study p_starve_ was never zero (see Table 1).

### Models

#### Overview

On average participants missed 2.0 trials of 960 trials in total (SD = 3.3). We determined best fitting model parameters using maximum likelihood estimation (MLE). Optimization used a non-linear Nelder-Mead simplex search algorithm (implemented in the MATALB function fminsearch) to minimize negative log-likelihood summed over all trials for each participant. We first ran optimization with positive seed parameters. In case of non-convergence, we seeded models with negative starting parameters. Log-likelihoods were obtained from the best fitting parameters. For each model we approximated model evidence by calculating the Bayesian Information Criterion (BIC), which penalizes model complexity (see Section 7 in S2 Text for results based on the Akaike Information Criterion, AIC).

All models used a logistic/softmax function with a free parameter β to generate trial-by-trial probabilities for choosing one of the two options, which allows for noise in action selection and is conceptually similar to logistic regressions.

$$P_{choose}=\frac{1}{1+e^{-(V_{1}-V_{2})/\beta }}$$ (1.1)

V_1_ and V_2_ are the values of the two options as specified by the models. The following description follows the order of the models as listed in Table 2. We initially devised nine models that were grouped into three model families.

In brief, the first two model families included variations of two types of economic models while the third model family comprised novel models based on homeostatic considerations. In the four models of the first family, the decision variable was based on different linear combinations of the statistical moments of the outcome distribution (expected value, EV; variance, Var; and skewness, Skw). The second family comprised two variants of rank-dependent utility models. In the three models of the third family, the decision variable was based on the probability of starvation (p_starve_) in addition to different linear combinations of statistical moments (see Table 2).

#### Model family 1: Moments without p_starve_

In the first family, models (Models 1–4) were based on the statistical moments of the outcome distribution. In the simplest model (Model 1) only the β parameter was included as a free parameter in order to compare the EVs between the two options and thus V simply equals the option's EV.

Model1:V=EV (1.2)

The three other models (Models 2–4) of the first family additionally included linear combinations of Var and Skw, which were each weighted by respective free parameters. That is, participants are allowed to express different preferences for these variables and each variable is weighted by a free parameter (ρ or λ).

Model2:V=EV+ρVar (1.3)

Model3:V=EV+λSkw (1.4)

Model4:V=EV+ρVar+λSkw (1.5)

#### Model family 2: Rank-dependent utility

The second family (Models 5–6) comprised two variations of rank-dependent utility models [23], in which the probabilities of the ranked outcomes were non-linearly weighted (and in which outcomes were transformed into utilities using an exponential function with one free parameter). Specifically, the foraging options contained *J* outcomes *x*_1_, *x*_2_,…, *x*_*J*_, with their respective probabilities *p*_1_, *p*_2_,…, *p*_*J*_. In the rank-dependent utility models, the options were evaluated according to

$$V=\sum_{j=1}^{J}\pi_{i}u(x_{j})$$ (1.6)

*u*(*x*_*j*_) is an increasing utility function over monetary outcomes with *u*(0) = 0. In line with many specifications in the literature we choose a power function with the free parameter μ.

$$u(x)=x^{\mu }$$ (1.7)

Decision weights *π*_*j*_ were defined depending on *w*(*p*_*j*_), which is an increasing probability weighting function with *w*(0) = 0, *w*(1) = 1, and $\sum_{i=1}^{J}\pi_{j}=1$.

$$\pi_{j}=\{\begin{array}{cc} w(p_{1}) & \text{for}\;j=1\\ w(\sum_{k=1}^{j}p_{k})-w(\sum_{k=1}^{j-1}p_{k}) & \;\text{for}\;i=2\leq j\leq J \end{array}$$ (1.8)

One of the two rank-dependent utility models (Model 5) used the weighting function specified by Prelec-I, which includes one free parameter α [23].

$$\text{Model}\;5:\;w\left(p\right)=\;\text{exp}\left(-{\left(-\text{log}\left(p\right)\right)}^{\alpha }\right)$$ (1.9)

The other of the two rank-dependent utility models (Model 6) used the weighting function specified by Prelec-II, which includes two free parameters and thus allows more flexible shapes than Prelec-I [23]. The additional free parameter is called β for consistency (not to be confused with the β of the logistic function).

$$\text{Model}\;6:\;w\left(p\right)=\;\text{exp}\left(-\beta {\left(-\text{log}\left(p\right)\right)}^{\alpha }\right)$$ (1.10)

#### Model family 3: Moments and p_starve_.

In the third family, models (Models 7–9) included a free parameter for p_starve_ (ξ) in addition to combinations of the statistical moments. The models of this third family thus included a free weighting parameter that is derived from homeostatic principles.

$$\text{Model}\;7:\;V=EV+\;\xi \;p_{starve}$$ (1.11)

$$\text{Model}\;8:\;V=EV+\rho Var+\xi \;p_{starve}$$ (1.12)

$$\text{Model}\;9:\;V=EV+\rho Var+\lambda Skw+\xi \;p_{starve}$$ (1.13)

#### Model comparison

We approximated model evidence using the Bayesian information criterion (BIC). Additionally, we report the Akaike information criterion (AIC). These were calculated for each participant and model according to the following equations.

BIC=-ln(L)+0.5k ln(n) (1.14)

AIC=-ln(L)+k (1.15)

where L is the likelihood function, k the number of parameters in the model and n the number of data points of the relevant participant. First, we performed random-effects analyses under the assumption that different participants may use different models or model families. We used the Bayesian Model Selection (BMS) procedure implemented in in the VBA-toolbox (http://mbb-team.github.io/VBA-toolbox) to calculate exceedance probabilities, which measure how likely it is that any given model is more frequent than all other models in the population [50–52]. We tested for the best fitting model family and subsequently for the best fitting model within the winning family. Second, we performed fixed-effects analyses, under the assumption that every participant uses the same model. We computed log Bayes factors by subtracting AIC/BIC for a reference model from the AIC/BIC of each tested model, and summed them over the group to yield log-group Bayes factors (see the main text for analyses based on BIC; see Section 7 of S2 Text and S1, S2, S4, and S5 Tables for analyses based on AIC). According to the convention used here, smaller log-group Bayes factors indicate more evidence for the respective model versus the baseline model. To compare parameter estimates, we used the exact method of the sign test as implemented in MATLAB.

#### Additional models

Since the model based on EV and p_starve_ (Model 7) was the overall winning model within the initial nine models, we adapted it and tested a tenth model (Model 10) which included two separate free parameters for p_starve_: foraging- p_starve_ and casino-p_starve_.

$$\text{Model}\;10:\;V=EV+f\;\xi_{foraging}p_{starve}\;+\left(\;1-f\;\right)\;\xi_{casino}p_{starve}$$ (1.16)

Where *f* = 1 for all choices in the foraging frame and *f* = 0 for all choices in the casino frame.

We further tested two supplementary models. The results related to these models are described in Sections 1 and 2 of S2 Text. Model 11 is a variant of Model 7 and Model 1, in which the option's value is simply given by the option's p_starve_ and not by its EV.

$$\text{Model}\;11:\;V=\;p_{starve}$$ (1.17)

Model 12 is a variant of Model 7, in which three separate free parameters are used for the three different numbers of foraging days in the gambles (corresponding to the number of options).

$$\text{Model}\;12:\;V=EV+\;d_{1}\xi_{d1}\;p_{starve}\;+\;d_{2}\xi_{d2}\;p_{starve}\;+\;d_{3}\xi_{d3}\;p_{starve}$$ (1.18)

Where *d*_1_ = 1 for all gambles with one day and *d*_2_ = 0 and *d*_3_ = 0 otherwise. Analogously, *d*_2_ = 1 for gambles with two days and *d*_3_ = 1 for gambles with three days.

#### Relationship between p_starve_ and skewness

We note that the model based on EV and p_starve_ (Model 7) outperformed models including a parameters that weighted the difference in skewness (Model 4, Model 9) even though differences in skewness and in starvation probability were correlated (Pearson’s r =. 901). While this correlation may have led to inflated errors of the parameter estimates in the model that included linear weighting factors for both p_starve_ and Skw (Model 9), it did not compromise model estimation, and thus model comparison, for the models that separately included weighting factors for either p_starve_ (Model 7, Model 8) or Skw (Model 3, Model 4). Also, within Model 9, variance inflation factors lay below 8 for all individuals.

### Analysis of RTs

Log-transformed RTs were analyzed using a linear mixed effects model as implemented in the R package lmer [53] (http://cran.r-project.org/web/packages/lme4/index.html). Log-transformed RTs were approximately normally distributed. The independent variables in the mixed effects model were the variables that the comparison of choice models identified as relevant (i.e., EV and p_starve_). Specifically, the fixed effects of the model included the difference between the two options in EV and p_starve_ as well as the interaction of the two. Random effects for participants included a random intercept and random slopes for EV, p_starve_, and their interaction. The model is given by the following equation:

$$\text{log}(RT)=\Delta EV+\Delta p_{starve}+\Delta EV*\Delta p_{starve}+(1+\Delta EV+\Delta p_{starve}+\Delta EV*\Delta p_{starve}|participant)$$ (1.19)

Significance levels of the fixed effects were determined by performing log-likelihood tests, which compared the full model to models without the respective factor.

## Supporting Information

S1 Text This document contains a detailed derivation of the mathematics of the random walks used to construct the gambles. (PDF)

S2 Text This document includes supporting results and supporting methods. (DOCX)

S1 Fig Choice and RT data binned according to differences in p_starve_ and EV. (A) We binned data according to the difference in p_starve_ between the two choice options and plot means across participants. Error bars indicate standard errors of the mean. Upper row: Since there was an uneven number of trials in the different bins, we provide histograms. Middle row: The higher the difference in p_starve_ between the two choice options was, the more likely participants chose the option with the lower value of p_starve_. The average slope was more negative in the foraging compared with the casino frame. Bottom row: Reaction times were modulated by p_starve_.
(B) We binned data according to the difference in EV between the two choice options. Conventions as in A. The higher the difference in EV between the two choice options was, the more likely participants chose the option with the higher EV.
(C) We binned data according to the differences in p_starve_ and in EV. To obtain a reliable number of trials in each bin, we only include the “middle” bins for the differences in p_starve_. For the differences in EV we performed a median split.
(TIF)

S1 Table Model family comparison: Relative log-group Bayes factors based on AIC. (DOCX)

S2 Table Model family comparison: Exceedance probabilities based on AIC. (DOCX)

S3 Table Model comparison for all data points: Individual model fits according to BIC. (DOCX)

S4 Table Comparison within the winning model family: Exceedance probabilities based on AIC. (DOCX)

S5 Table Comparison of an additional model including frame-specific parameters: Relative log-group Bayes factors and exceedance probabilities based on AIC. (DOCX)

S6 Table Comparison of a model based solely on EV with a model based solely on p_starve_. (DOCX)

S7 Table Comparison of a model with day-specific weighting parameters for p_starve_. (DOCX)

S8 Table Comparison of models based on the assumption that participants falsely included values below zero in the outcome distributions in the foraging frame. (DOCX)

S1 Dataset This file contains the behavioral data of all subjects. (MAT)
