# Supplementary material for: Maintaining Homeostasis by Decision-Making
Source: PLoS Comput Biol. 2015 May 29;11(5):e1004301. doi: 10.1371/journal.pcbi.1004301 (PMC4449003; doi:10.1371/journal.pcbi.1004301)
Supplement: S1 Text — (PDF) [file pcbi.1004301.s001.pdf]

S1 Text: Maintaining Homeostasis by Decision-Making

April 30, 2015

1 Random walk to generate gambles for virtual foraging task

1.1 General idea

We propose that decision making aimed at maintaining homeostasis (i.e., aimed at avoiding to die from hunger) can be investigated by using gambles that are derived within the mathematical framework of random walks.

- To maintain homeostasis, a biological agent has to keep its internal energy resources or energy points x above zero at any (discrete) time point i (with $x,\ i \in \mathbb{N}_0$).
- In each trial (i.e., at each new foraging decision), the agent starts (the random walk) with internal resources x_0 at time point $i = 0$. Within each trial, the agent passes through n time steps (i.e., "days").
- At each time step $n = 1$, the agent's internal resources incur a sure cost$-c$ (with $c \in \mathbb{N}_0$), which mirrors the consumption of energy (e.g., in terms of calories).
- To replenish the internal resources, the agent choses a risky foraging option and probailistically receives its outcomes at each time step n. That is, within a given time step $n = 1$ the agent can gain an amount g (with $g \in \mathbb{N}_0$) with probability p. This would, for example, correspond to collecting berries or to hunting deer, where berries could provide a lower gain (less calories) but have a higher probability than deer. The probabilities of finding berries or hunting down deer could, for example, vary according to different seasons or environments.
- Alternatively, if the agent does not gain anything, the internal resources only incur the sure cost$-c$ with probability q (where $q = 1 - p$).
- The gain is assumed to be equal or larger than the cost, otherwise the agent would not strive for it, therefore $g \geq c$.

This situation represents a random walk starting at x_0 with

- a step size of $g - c$ and a probability p of going right and
- a step size of$-c$ and a probability q of going left.

The random walk has a lower absorbing barrier at $x_b = 0$, which mirrors dying from hunger. Within this framework, gambles can be constructed as a function of p (and thus q), n, x_0, c, and g.

1.2 Assumptions

A number of simplifications are made:

1. The agent makes one single decision at the beginning of each trial (e.g., the agent decides whether to collect berries or to hunt deer and sticks to that decision throughout the number of days). That is, the gambles consist of compound lotteries, which comprise n sequential lotteries.
2. The agent does not deplete the food sources and does not get more proficient at obtaining food. That is, the probabilities p and q are constant within each trial.
3. Similarly, cost c and gain g are constant within each round.
4. Costs do not differ between foraging options. For example, collecting berries and hunting deer are assumed to have the same costs (in terms of calories spent).
5. Here, only a lower absorbing barrier is considered. An upper absorbing barrier (e.g., death due to overeating) is not included.
6. Dying from hunger represents the only threat to homeostasis (e.g., there are no predators).
7. Only a single variable (the amount of internal energy resources) has to be kept within a homeostatic range (e.g., there is no need to obtain specific nutrients and no conflicts or opportunity costs with respect to other activities such as sleep or reproduction).

1.3 Outcome distributions of the random walk

In the following, the probability distributions of random walks will be described. The description of random walks will increase in complexity until all features outlined above can be incorporated.

1.3.1 Simple random walk starting at zero

A random walk is called *simple* if steps to the right have the step size $+1$ and steps to the left have the step size -1 (i.e., if $c = 1$ and $g = 2$; since $g - c$ is the step size to the right and since$-c$ is the step size to the left). Let's assume the agent starts the random walk at zero. Let X_n denote the random variable which indicates the position of the agent after n time steps. Then,

$$p = P(X_1 = 1)$$
$$q = 1 - p = P(X_1 = -1)$$

Let W_n denote the number of steps to the right within the first n steps. Then W_n has a binomial distribution.

$$P(W_n = a) = \binom{n}{a} p^a \, q^{n-a}, \quad a \in \mathbb{N}_0.$$

If there are a steps to the right $(+1)$ and therefore $n - a$ steps to the left (-1) then

$$X_n = a(+1) + (n - a)(-1) = 2a - n.$$

If n is even then X_n is also even and if n is odd then X_n is also odd. Therefore, if n and x are not either both even or both odd then $P(X_n = x) = 0$. Put differently, the range of X_n is

$$X_n = \{-n, -n+2, -n+4, \ldots, n-4, n-2, n\}.$$

After each number of steps n there are $n + 1$ possible positions. That is, the range of X_n contains $n+1$ elements. If the agent has visited a certain position at a certain time step n, the agent can visit it again at $n+2$, $n+4$, ... time steps. Another way of saying this is that if x and n are both even or odd $P(X_n = x) \geqq 0$, otherwise $P(X_n = x) = 0$. $W_n = a$ if and only if $X_n = 2a - n$. Writing $x = 2a - n$, so that $a = \frac{n+x}{2}$ and $n - a = \frac{n-x}{2}$, the probability distribution of X_n is

$$P(X_n = x) = \binom{n}{\frac{n+x}{2}} p^{\frac{n+x}{2}} \, q^{\frac{n-x}{2}}.$$

1.3.2 Simple random walk starting at position x_0

Now, let's assume that the agent does not start the random walk at zero but at x_0. Let $X_n(x_0)$ denote the random variable which indicates the position of the agent after n time steps in a simple random walk starting from x_0. Then,

$$p = P(X_1 = x_1 = x_0 + 1)$$
$$q = 1 - p = P(X_1 = x_1 = x_0 - 1)$$

and

$$X_n(x_0) = a(+1) + (n-a)(-1) + x_0 = 2a - n + x_0.$$

The range of $X_n(x_0)$ is

$$\begin{aligned} X_n(x_0) = \{ & -n + x_0, -n + x_0 + 2, -n + x_0 + 4, ..., \\ & n + x_0 - 4, \, n + x_0 - 2, \, n + x_0 \} . \end{aligned}$$

Writing $x = 2a - n + x_0$, so that $a = \frac{n+x-x_0}{2}$ and $n - a = \frac{n-x+x_0}{2}$, the probability distribution of $X_n(x_0)$ is

$$P(X_n(x_0) = x) = \binom{n}{\frac{n+x-x_0}{2}} p^{\frac{n+x-x_0}{2}} q^{\frac{n-x+x_0}{2}}.$$

1.3.3 Random walk with unequal step sizes

Now, let's assume that step sizes to the right and step sizes to the left can take values $\neq \pm 1$. Steps to the right have the size $g - c$ and steps to the left have the size $-c$. Then,

$$\begin{aligned} p &= P(X_1(x_0) = x_1 = x_0 + g - c) \\ q = 1 - p &= P(X_1(x_0) = x_1 = x_0 - c) \end{aligned}$$

and

$$X_n(x_0) = a(g-c) + (n-a)(-c) + x_0 = ag - nc + x_0.$$

The range of $X_n(x_0)$ is

$$\begin{aligned} X_n(x_0) = \{ & -nc + x_0, -nc + x_0 + g, -n + x_0 + 2g, ..., \\ & (g-c)n + x_0 - 2g, \, (g-c)n + x_0 - g, \, (g-c)n + x_0 \} . \end{aligned}$$

If the agent has visited a certain position at a certain time step n, the agent can visit it again at $n+g$, $n+2g$, $\ldots$ time steps. Writing $x = ag - nc + x_0$, so that $a = \frac{nc+x-x_0}{g}$ and $n - a = \frac{n(g-c)-x+x_0}{g}$, the probability distribution of $X_n(x_0)$ is

$$P(X_n(x_0) = x) = \binom{n}{\frac{nc+x-x_0}{g}} p^{\frac{nc+x-x_0}{g}} q^{\frac{n(g-c)-x+x_0}{g}}.$$

1.3.4 Absorbing barrier at zero

So far the random walk has been unrestricted. That is, it had no (absorbing) barrier (and thus dying was not possible). In a random walk with an absorbing barrier at zero $x_b = 0$ the range of $X_n(x_0)$ only includes elements ≥ 0. Conceptually, if one imagines the random walk as a tree, in which new branches are added at each time step n, one has to first calculate the probability distribution within the full tree (i.e., without considering the barrier). Then, to calculate the probability distribution of a random walk with an absorbing barrier at zero,

all downstream branches starting from visits at zero (or values below zero) have to be "pruned" (i.e., subtracted) from the full tree, which is given by the corresponding random walk without absorbing barrier.

To calculate the probabilities of starvation, i.e. the probabilities of reaching zero (or values below zero) within n time steps, one has to perform the following three steps.

1. Determine all time steps h_i when "hits" of the barrier can occur (i.e., time steps n in which the agent in an unrestricted walk can be at $x_b = 0$; with $h \leq n$; $i < n$).

2. For all those hits of the barrier h_i, calculate the probabilities of the agent being at $x_b = 0$ for the first time.

3. Add up the probabilities of the agent being at $x_b = 0$ for the first time at all time steps h_i when hits of the barrier occur.

(Note that for step sizes to the left unequal to $\neq -1$, analogous calculations have to be made for all cases in which the agent can be at a position $x < 0$ without passing through zero. E.g. if $c = 2$, the random walk can go directly from $+1$ to -1. For simplicity these cases are not explicitly described below but the rationale is the same.)

Hits of the barrier Determine the hits of the barrier h_i within n time steps for all i (i.e., the first time that the range of $X_n(x_0)$ includes zero). The agent can visit the barrier x_b at h_1, h_1+g, h_1+2g, h_1+3g, ... time steps until $h_i \leq n$.

Probabilities of hitting the barrier for the first time The probability for the agent in a random walk (starting from x_0) to be at $x_b = 0$ for the first time after n steps is defined as

$$f_n(x_0) = P(X_n(x_0) = x_b)$$

and

$$X_r(x_0) \neq x_b, \quad 0 < r < n.$$

To calculate $f_n(x_0)$, one needs to calculate the probability for the agent in a random walk (starting from x_0) to be at $x_b = 0$ *not necessarily for the first time* after n steps, which is defined as

$$u_n(x_0) = P(X_n(x_0) = x_b)$$

There is no explicit formula for $f_n(x_0)$ for random walks with unequal step sizes and an absorbing barrier. But $f_n(x_0)$ can be found as a function of $u_n(x_0)$. At the first hit of the barrier h_1

$$f_{h_1}(x_0) = u_{h_1}(x_0)$$

To find the probability of hitting the barrier for the first time at h_2, let's consider the example of a simple random walk starting at one (i.e., $x_0 = 1$). The agent

can hit $x_b = 0$ after 1, 3, 5,... time steps (i.e., h_1, $h_1 + g$, $h_1 + 2g$, ... time steps; and thus $h_1 = 1$, $h_2 = 1 + g$, $h_3 = 1 + 2g$, ...). There are two mutually exclusive ways in which the agent can be at $x_b = 0$ at the second hit of the barrier $h_2 = 3$.

- First, the agent visits zero for the first time at h_2 (i.e., at $h_2 = 3$ with probability $f_{h_2}(x_0)$).
- Second, the agent repeatedly visits zero. That is, the agent visits zero for the first time at h_1 (i.e., at $h_1 = 1$ with probability $f_{h_1}(x_0) = u_{h_1}(x_0)$) *and returns to zero* after $g = 2$ further time steps. That is, the probability of going from the first hit of the barrier h_1 to the second hit of the barrier h_2 within $g = 2$ time steps is $u_g(x_b)$. Therefore, the probability of having repeatedly visited zero at h_2 is

$$f_{h_1}(x_0)\, u(x_b).$$

Since the first and the second way of reaching zero are mutually exclusive, they can be added up. Therefore, at the second hit of the barrier h_2

$$u_{h_2}(x_0) = f_{h_2}(x_0) + f_{h_1}(x_0)u_g(x_b)$$

and

$$f_{h_2}(x_0) = u_{h_2}(x_0) - f_{h_1}(x_0)u_g(x_b).$$

More generally, there are i mutually exclusive ways in which the agent can hit the barrier $x_b = 0$ at h_i (with probability $u_{h_i}(x_0)$).

- First, the agent visits the barrier x_b for the first time at time step h_i (with probability $f_{h_i}(x_0)$).
- Second, the agent repeatedly visits the barrier. That is, the agent visits the barrier for the first time at one of the previous time steps $h_{i-1}, h_{i-2}, h_{i-3}$, ... , h_1(with probabilities $f_{h_{i-1}}(x_0)$, $f_{h_{i-2}}(x_0)$, $f_{h_{i-3}}(x_0)$, ... , $f_{h_1}(x_0) = u_{h_1}(x_0)$) *and returns to the barrier* after g, $2g$, $3g$, ... , $(i-1)g$ further time steps.

  That is, the probabilities of going from the hits of the barrier $h_{i-1}, h_{i-2}, h_{i-3}$, ... , h_1 to the hit of the barrier h_i are $u_g(x_b), u_{2g}(x_b), u_{3g}(x_b)$, ... , $u_{(i-1)g}(x_b)$.

  Therefore, the probability of having repeatedly visited the barrier $x_b = 0$ at h_i is

$$f_{x_0,h_{i-1}}u_{0,g} + f_{x_0,h_{i-2}}u_{0,2g} + f_{x_0,h_{i-3}}u_{0,3g} + \ldots + f_{x_0,h_1}.$$

Therefore,

$$f_{h_i}(x_0) = u_{h_i}(x_0) - f_{h_{i-1}}(x_0)u_g(x_b) - f_{h_{i-2}}(x_0)u_{2g}(x_b) - \ldots - f_{h_i}(x_0).$$

Note that the values of the indices within a product have to add up to h_i. Note also that the range of X_n contains $n + 2 - i$ elements for $i \geqq 1$ (where i are the number of hits of the barrier).

Adding up probabilities of hitting the barrier for the first time To find the probability of having reached the absorbing barrier at $x_b = 0$ within n time steps (i.e., the probability of starvation p_{starve}), add up the probabilities of being at $x_b = 0$ for the first time at all possible hits h_i of the barrier.

$$P(X_n(x_0) = 0) = f_{h_i}(x_0) + f_{h_{i-1}}(x_0) + \ldots + f_{h_1}(x_0)$$

2 Calculating statistical moments

2.1 Expected value

To calculate the first statistical moment, i.e., the expected value of a random walk with an absorbing barrier x_b (at zero) at time step n, one has to first calculate the probabilities of all elements $P(X_n(x_0) = x)$ within the range of $X_n(x_0)$ and then take the sum of all those probabilities multiplied by their respective values $P(X_n(x_0) = x)\,x$.

The rationale is similar as above for finding the probabilities of hitting the absorbing barrier. To find the probabilities of being at a certain position x in a random walk with an absorbing barrier (at zero), one has to first calculate the probability distribution within the corresponding full tree (i.e., without considering the barrier). Then, all downstream branches starting from visits at zero (or values below zero) have to be "pruned" (i.e., subtracted) from the full tree. The time steps when the random walk can hit zero h_i and the probabilities when the random walk reaches zero $f_{h_i}(x_0)$ have already been determined above.

- First, calculate the probability of being in x in the corresponding full tree without absorbing barrier P_{full}. (This probability corresponds to $u_{h_i}(x_0)$ above.)
$$P_{full}(X_n(x_0) = x)$$
- Second, calculate the downstream branches. That is, the probabilities of going from the barrier x_b to x (at all time steps h_i when the barrier was hit). (These probabilities correspond to $u_g(x_b)$, $u_{2g}(x_b)$, $u_{3g}(x_b), \ldots,$ $u_{(i-1)g}(x_b)$ above.)

$$P(X_{n-h_i}(x_b) = x), P(X_{n-h_{i-1}}(x_b) = x), P(X_{n-h_{i-2}}(x_b) = x), \ldots, P(X_{n-h_1}(x_b) = x)$$

The probability of being in position x in a tree with an absorbing barrier at $x_b = 0$ is

$$\begin{aligned} P_b(X_n(x_0) = x) = & P_{full}(X_n(x_0) = x) - f_{h_i}(x_0)P(X_{n-h_i}(x_b) = x) + \\ & - f_{h_{i-1}}(x_0)P(X_{n-h_{i-1}}(x_b) = x) - \ldots - f_{h_1}(x_0)P(X_{n-h_1}(x_b) = x). \end{aligned}$$

The expected value (EV) is the weighted sum over all J elements $x_1, x_2, \ldots, x_J$ within the range of $X_n(x_0)$. The number of elements J is $n + 2 - i$ for $i \geqq 1$ (where i are the number of hits of the barrier; for $i = 0$, i.e., no hits of the barrier, J equals $n + 1$).

$$EV = \sum_{j=1}^{J} P(X_n(x_0) = x_j)\, x_j$$

2.2 Variance and skewness

The second and third statistical moments, i.e., variance (Var) and skewness (Skw) are calculated as follows

$$Var = \sum_{j=1}^{J} P(X_n(x_0) = x_j)\,(x_j - EV)^2$$

$$Skw = \frac{\sum_{j=1}^{J} P(X_n(x_0) = x_j)\,(x_j - EV)^3}{Var^{\frac{3}{2}}}$$
