# Supplementary material for: Maintaining Homeostasis by Decision-Making
Source: PLoS Comput Biol. 2015 May 29;11(5):e1004301. doi: 10.1371/journal.pcbi.1004301 (PMC4449003; doi:10.1371/journal.pcbi.1004301)
Supplement: S2 Text — (DOCX) [file pcbi.1004301.s002.docx]

**S2 Text: Maintaining Homeostasis by Decision-Making**

**Supporting results**

1. **Model including only a free parameter for p_starve_**

To address whether p_starve_ or EV contributed more to participants’ decisions, we also devised a model in which the decision probability was determined solely by a sigmoid transformation of the difference in p_starve_ (but not of the difference in EV; Model 1). Taking both frames into account, this model performed slightly better than the model which only included EV as shown by fixed- and random-effects analyses (see S6 Table). This suggests that overall p_starve_ contributed more to participants’ choices than EV. When fitting this model separately to the foraging and the casino frames, the model that only included p_starve_ explained choices better in the foraging frame but the model that only included EV explained choices better in the casino frame. These results suggest that p_starve_ seemed to have a greater influence in the biological context of the foraging frame and EV seemed to play a comparatively greater role in the purely economic context of the casino frame. This pattern complements the finding that in the model that included frame-specific weighting parameters for the parameter estimates for p_starve_ (Model 10) participants minimized p_starve_ more in the foraging than in the casino frame.

1. **Model testing for the effect of the number of foraging days**

The gambles in the foraging frame comprised a different number of days, which corresponded to a different number of possible outcomes in the foraging and equivalently in the casino frame. Possibly, the different number of possible outcomes could translate into different weightings of p_starve_. For example, the increasing levels of complexity due to the increasing number of possible outcomes might make participants more conservative, resulting in a more pronounced minimization of p_starve_. To test this possibility, we devised a model, in which we included three different weighting parameters (ξ_d1_, ξ_d2_, and ξ_d3_) for p_starve_ in the gambles with 1, 2, or 3 foraging days. We compared this model to the model that was based on EV and p_starve_ globally (Model 7). Fixed- and random-effects analyses showed that the simpler model outperformed the more complex model that included day-specific weighting parameters (see S7 Table). Thus, the conjecture that different numbers of possible outcomes led to different weightings of p_starve_ could not be supported.

1. **Detailed comparison of parameter estimates between the two frames**

In the main text, we showed that across participants ξ_foraging_ was smaller than ξ_casino_. We confirmed that this difference was not driven by an outlier participant for whom ξ_casino_ was more than 3 SD higher than the mean. The difference remained significant after excluding this participant (p<.05). A similar pattern emerged when fitting the winning model (Model 7) separately to the foraging and casino frames (and separately to the first and second blocks): All weighting parameters of p_starve_ were significantly smaller than zero across participants (sign test on parameters: foraging both blocks ξ: p<.001; casino both blocks ξ: p<.005; foraging-block 1 ξ: p<.001; foraging-block 2 ξ: p<.001; casino-block 1 ξ: p<.005; casino-block 2 ξ: p<.005). In models of foraging frame weighting parameters were smaller than in models of the casino frame. This effect reached trend level when including data from both blocks (p=.053) or block 1 (p=.053). For block 2 the effect was significant (p<.05).

We note that the options in casino frame were all presented as single-step gambles whereas the options in the foraging frame were presented as sequential gambles. Thus, both the difference between single-step and sequential gambles, and the different framing, could potentially underlie the smaller parameter estimates for p_starve_ in the foraging frame. To disambiguate these possibilities, we directly compared gambles in foraging frame with a single foraging day (i.e., single-step foraging gambles) with the corresponding single-step casino gambles (120 gambles per frame) in Model 10. We still found a trend for ξ_foraging_ being smaller than ξ_casino_ (sign test comparing ξ_foraging_ and ξ_casino_: p=.053). We also fitted the winning model (Model 7) separately to the foraging and the casino frames gambles and only included single-step gambles. ξ_foraging_ was significantly smaller ξ_casino_ (p<.05). These results suggest that the difference between sequential and single-step presentation was not the only factor driving participants’ stronger minimization of p_starve_ in the foraging versus the casino frame. Instead, framing per se seems to impact the degree of p_starve_-minimization.

1. **Comparison of parameter estimates for different energy levels and number of days**

Participants did not receive feedback on the outcomes in the task. This also entails that they did not see the intermediate outcomes in the foraging task (i.e., the outcomes after each foraging day). Additionally, the number of foraging days was related to the number of initial energy points in the gambles used in the present study. Since we wanted p_starve_ to be non-zero in all gambles, the number of energy points was always 1 in options with 1 foraging day. The lack of feedback and the relation of the number of foraging days to initial energy levels could have resulted in a superposition of opposing effects: Participants could focus on p_starve_ differently depending on the number of days and/or the initial foraging level. To test this, we compared the parameter estimates for p_starve_ when including different types of trials within the winning model (Model 7).

We fitted models separately to options with (a) an energy level of 1 and 1 foraging day (120 trials per frame), (b) an energy level of 1 and 2 foraging days (120 trials per frame), and (c) an energy level of 2 and 2 or 3 foraging days (240 trials per frame). Within the foraging frame, we did not find evidence for differences in the magnitude of the parameter estimates when performing pairwise comparison across participants using sign tests (all p’s>0.2, even without correction for multiple comparisons). The same was true in the casino frame (all p’s>0.2). When additionally splitting the options with an energy level of 2 into those with 2 and those with 3 foraging days, the same pattern emerged. Neither in in the foraging frame nor in the casino frame any pairwise comparison reached significance (all p’s>0.05, even without correction for multiple comparisons).

These supplemental analyses suggest that within the gambles used in the current study the magnitude of p_starve_ was not different for different combinations of energy levels and foraging days.

1. **Testing for the use of outcome distributions including values below zero in the foraging frame**

To mirror starvation, the outcome distributions of the options used in the task did not include values below zero and participants were explicitly instructed about this. Nevertheless, in the foraging frame it is possible that participants did not correctly “prune” the values below zero in their estimation of the outcome distributions and may have included them in the approximation of the statistical moments (e.g., in the “tree” depicted in Fig 1C, participants could erroneously include an outcome of -1). In the casino frame, the outcome distributions were explicitly signaled using pie charts and therefore participants had no reason at all to falsely include values below zero. Erroneously including values below zero could theoretically mimic an (enhanced) behavioral weight of p_starve_. The results of the following two different types of analyses make this unlikely.

First, in the options used in the current study negative outcomes could be falsely estimated for options with an energy level of 1 and 2 foraging days and for options with energy level of 2 and 3 days but not otherwise. In the other options the lowest outcome was zero (i.e., energy level of 1 and 1 day; energy level of 2 and 2 days). In the previous section, we reported that the magnitude of p_starve_ did not differ between the four different combinations of energy levels and foraging days employed, which indicates that the weighting of p_starve_ did not depend on the inclusion of values below zero.

Second, we calculated the outcome distributions that included values below zero for all options and derived the statistical moments for these distributions. We then compared the models of model families 1 and 3 since these two families were based on statistical moments. Random- and fixed-effects analyses showed that family 3 provided the best fit—even under the assumption that participants erroneously included values below zero in their estimation of the outcome distributions in the foraging frame (S8 Table).

Taken together, two types of supplemental analyses showed that participants’ reliance of p_starve_ cannot be explained by the possibility that participants erroneously included values below zero in the outcome distributions.

1. **Detailed comparison between the two blocks of the foraging frame**

We note that the model comparisons were not as decisive when only considering data from foraging-block 2 (see Tables 2-4) compared to when considering all data or data from foraging-block 1. Foraging-block 2 was the last block that participants performed and therefore participants may have been less attentive, which could translate into higher parameter estimates for decision noise. We indeed found a trend for higher parameters estimates of decision noise in foraging-block 2 versus foraging-block 1 (t(21)=-1.83: p=.082). Importantly, we found no significant difference in parameters of p_starve_ between foraging-blocks 1 and 2 (p=.16), which makes it unlikely that participants weighted p_starve_ less in foraging-block 1 than in foraging-block 2 (i.e., it is unlikely that participants become less reliant on the probabilities of the zero outcomes).

1. **Model comparisons based on AIC**

The model comparisons reported in the main text were based on BIC. In addition, we performed all model comparisons on the basis of AIC, which penalizes model complexity less severely than BIC in our data. Overall, the results of the model comparisons were consistent for BIC and AIC (see S1, S2, S4, and S5 Tables). We note two differences. First, the overall fixed-effects analysis using AIC tended to favor family 2 instead of family 3 (S1 Table) but the random-effects analysis still favored family 3 albeit not strongly (S2 Table). This effect was driven by the casino frame and in particular the first block of the casino frame. Nevertheless, in line with the BIC analyses, the overall winning model (Model 10), which included two frame-specific weighting parameters for p_starve_, outperformed both models of family 2 (Models 5 and 6) by a large margin when performing fixed-effects analyses based on AIC (Model 5: -2261; Model 6: -2308; Model 10: -3094). This was the case even though Model 10 contains less parameters than Model 5 and the same number of parameters as Model 6 (Model 5: 3 parameters; Model 6: 4; Model 10: 3). Second, in the comparison within model family 3, AIC favored the more complex model that included weighting parameters for Var and Skw in addition to a weighting parameter for p_starve_ (Model 9) while BIC favored the simpler model that only included a weighting parameter for p_starve_ (Model 7; see Tables 4 and S4 Table). We stress that the BIC is commonly regarded as the more principled measure and that these differences merely concern details of the specific overall winning model but do not invalidate our main result that models based on homeostatic considerations explained participants' choices over and above standard economic models.

1. **Questionnaire analyses**

For exploratory analyses, we also obtained participants’ meta-cognitive assessments of real-life risks in different domains. Specifically, we explored whether the parameter estimates derived from Model 10 for ξ_foraging_ or ξ_casino_ correlated with the relevant subscores on the DOSPERT scale. However, this was not the case (all p’s>.05, even without correction for multiple comparisons) and thus at present we do not make claims about how behavior in our task translates into meta-cognitive assessments of real-life risks.

**Supporting methods**

**Additional task description—foraging frame**

The depiction of the foraging frame comprised three components: An energy bar, a number of days, and two foraging options with probabilistic costs and gains. Participants had to decide between the two foraging options, which were depicted as pie charts with two sectors. One sector showed the probability of successful foraging with the associated variable amount of energy points to gain and the other sector showed the probability of unsuccessful foraging, which was always associated with a gain of zero energy points. Participants were told that for each day the chosen foraging option was played out (i.e., the respective points to gain were added to the energy bar according to the probabilities depicted in the pie chart). In addition, one energy point was deducted from the energy bar on each day to mirror energy consumption. If, at any point, the energy bar reached zero points, the participant had died from starvation within that trial and would thus receive no money. Otherwise, the final number of points would be exchanged for money. Participants did not see the outcomes of their choices. In each trial, participants first saw the energy bar and the number of days for 1 s and then had a maximum of 8 s to decide between the left and right option, which were counterbalanced for position. Their choice was indicated with an asterisk for 0.5 s and after a fixation cross of 0.75 s the next trial started.

**Additional task description—casino frame**

In the casino frame, participants had to decide between two wheel-spinning-like gambles that were depicted as pie charts with two to four sectors depicting the probabilities of different amounts to gain. Probabilities and amounts corresponded to the possible outcomes in the foraging frame. The number of outcomes in the foraging frame depended on the number of days. Gambles with one day had two outcomes and each additional day resulted in one additional outcome. The sectors were ordered according to ascending amounts to gain in anti-clockwise fashion. The lowest amount to gain was always zero, and its probability corresponded to p_starve_ in the foraging frame. Otherwise the casino frame was similar to the foraging frame: Points were to be exchanged for money. No outcomes were revealed. Left and right options were counterbalanced for position. Participants had up to 6 s to decide. Their choice was indicated with an asterisk for 0.5 s and after a fixation cross of 0.75 s the next trial started.

**Questionnaire**

To enable us to explore potential relationships between behavior in our task and participants’ meta-cognitive assessments of real-life risk, they completed a 30 item version of the domain-specific risk-taking (DOSPERT) scale [1] at the end of the experimental session. The DOSPERT scale assesses (a) participants’ tendencies to engage in risky behaviors, (b) their perceptions of risk, and (c) their expected benefits from risky behavior in six different domains, namely investing, gambling, health, recreational, ethical, and social. Since the domains investing, gambling, health and recreational were most closely related to our task, we correlated these subscores with parameter estimates derived from the overall winning behavioral model Model 10 (i.e., parameter estimates for ξ_foraging_ or ξ_casino_).

**Supporting references**

1. Johnson JG, Wilke A, Weber EU. Beyond a trait view of risk taking: A domain-specific scale measuring risk perceptions, expected benefits, and perceived-risk attitudes in German-speaking populations. Polish Psychol Bull. 2004;35(3):153–63.
