# Supplementary figures and images for: Maintaining Homeostasis by Decision-Making
Source: PLoS Comput Biol. 2015 May 29;11(5):e1004301. doi: 10.1371/journal.pcbi.1004301 (PMC4449003; doi:10.1371/journal.pcbi.1004301)

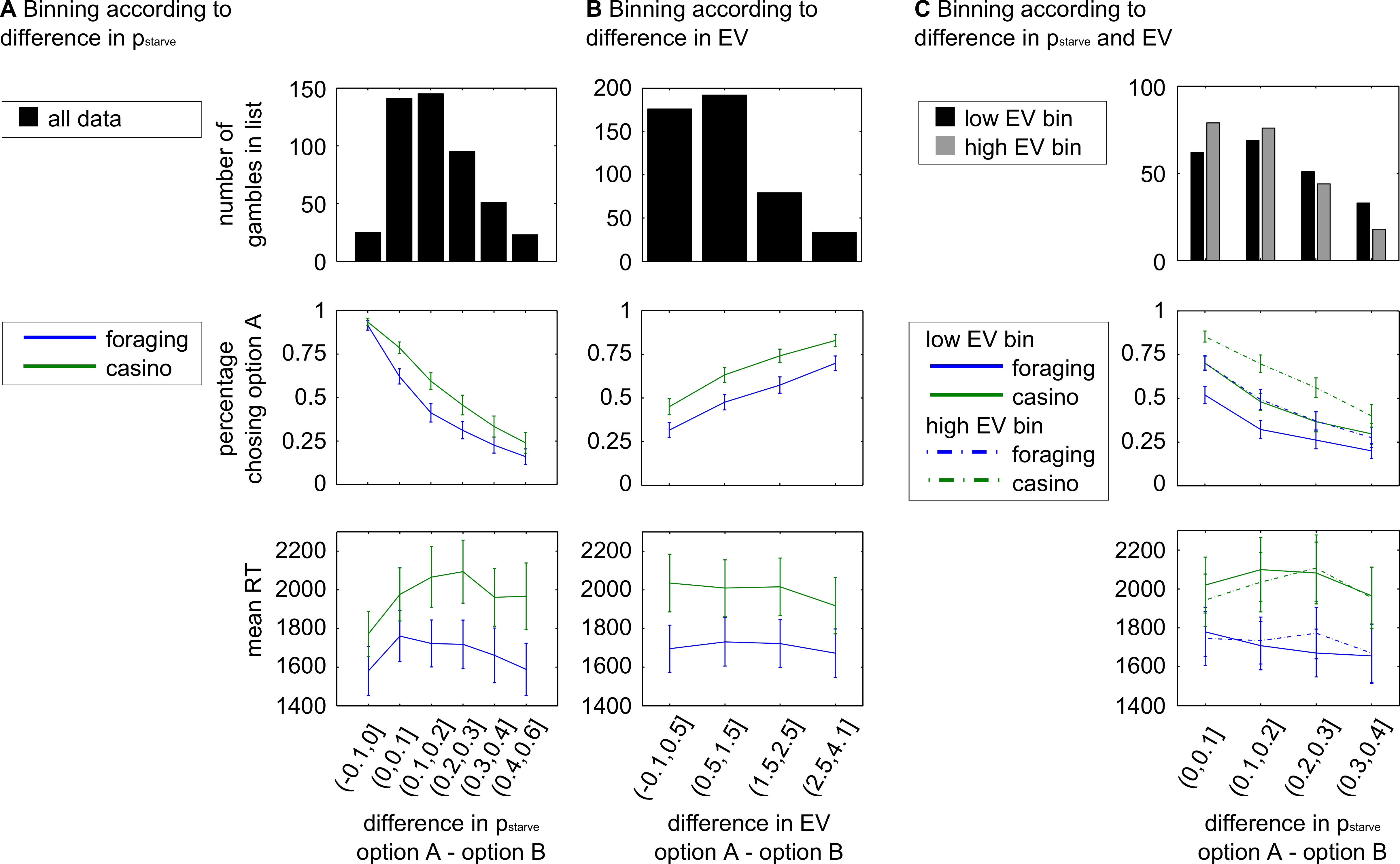

Supplement: S1 Fig — (A) We binned data according to the difference in pstarve between the two choice options and plot means across participants. Error bars indicate standard errors of the mean. Upper row: Since there was an uneven number of trials in the different bins, we provide histograms. Middle row: The higher the difference in pstarve between the two choice options was, the more likely participants chose the option with the lower value of pstarve. The average slope was more negative in the foraging compared with the casino frame. Bottom row: Reaction times were modulated by pstarve. (B) We binned data according to the difference in EV between the two choice options. Conventions as in A. The higher the difference in EV between the two choice options was, the more likely participants chose the option with the higher EV. (C) We binned data according to the differences in pstarve and in EV. To obtain a reliable number of trials in each bin, we only include the “middle” bins for the differences in pstarve. For the differences in EV we performed a median split. (TIF) [file pcbi.1004301.s003.tif]
