# Supplementary material for: Maintaining Homeostasis by Decision-Making
Source: PLoS Comput Biol. 2015 May 29;11(5):e1004301. doi: 10.1371/journal.pcbi.1004301 (PMC4449003; doi:10.1371/journal.pcbi.1004301)
Supplement: S1 Table — (DOCX) [file pcbi.1004301.s004.docx]

**S1 Table.** Model family comparison: relative log-group Bayes factors based on AIC

|  | Relative log-group Bayes factors based on AIC (smaller is better) | | | | | | | | |
| --- | --- | --- | --- | --- | --- | --- | --- | --- | --- |
|  | Family 1 | | | | Family 2 | | Family 3 | | |
|  | Moments without p_starve_ | | | | Rank-dependent utility | | Moments and p_starve_ | | |
|  | Model | Model | Model | Model | Model | Model | Model | Model | Model |
|  | 1 | 2 | 3 | 4 | 5 | 6 | 7 | 8 | 9 |
|  | EV | EV | EV | EV | Prelec-I | Prelec-II | EV | EV | EV |
|  |  | Var | Skw | Var |  |  | p_starve_ | Var | Var |
|  |  |  |  | Skw |  |  |  | p_starve_ | Skw |
|  |  |  |  |  |  |  |  |  | p_starve_ |
| All | 0 | -1387 | -2037 | -2139 | -2261 | **-2308** | -2244 | -2274 | -2287 |
| Foraging | 0 | -930 | -1435 | -1529 | -1557 | -1528 | -1557 | -1624 | **-1707** |
| Casino | 0 | -755 | -991 | -1059 | -1132 | **-1161** | -1107 | -1136 | -1108 |
| Foraging-block 1 | 0 | -511 | -721 | -772 | -810 | -794 | -820 | -827 | **-855** |
| Foraging-block 2 | 0 | -450 | -700 | -806 | -802 | -804 | -789 | -814 | **-888** |
| Casino-block 1 | 0 | -402 | -490 | -532 | -565 | **-580** | -556 | -563 | -554 |
| Casino-block 2 | 0 | -380 | -557 | -583 | -601 | -578 | -603 | **-613** | -602 |

For a fixed-effects analysis, log-group Bayes factors based on AIC were calculated relative to the simplest model (Model 1). Smaller log-group Bayes factors indicate more evidence for the respective model versus the baseline model. The log-group Bayes factors of the winning models according to fixed-effects analyses are written in bold font. The models included free parameters for the respective variables listed. See Table 2 for results based on BIC. AIC, Akaike information criterion; EV, expected value; Var, variance; Skw, skewness; p_starve_ starvation probability; BIC, Bayesian information criterion
