# Supplementary material for: Maintaining Homeostasis by Decision-Making
Source: PLoS Comput Biol. 2015 May 29;11(5):e1004301. doi: 10.1371/journal.pcbi.1004301 (PMC4449003; doi:10.1371/journal.pcbi.1004301)
Supplement: S2 Table — (DOCX) [file pcbi.1004301.s005.docx]

**S2 Table.** Model family comparison: exceedance probabilities based on AIC

|  | Exceedance probabilities based on AIC (higher is better) | | |
| --- | --- | --- | --- |
|  | Family 1 | Family 2 | Family 3 |
|  | Moments without p_starve_ | Rank-dependent utility | Moments and p_starve_ |
| All | 0.0010 | 0.3599 | **0.6391** |
| Foraging | 0.0002 | 0.0079 | **0.9919** |
| Casino | 0.0008 | **0.9036** | 0.0956 |
| Foraging-block 1 | 0.0017 | 0.0047 | **0.9936** |
| Foraging-block 2 | 0.0110 | 0.0314 | **0.9576** |
| Casino-block 1 | 0.0007 | **0.7672** | 0.2321 |
| Casino-block 2 | 0.0552 | 0.1230 | **0.8218** |

The highest exceedance probabilities according to random-effects analyses are written in bold font. See Table 3 for results based on BIC. AIC, Akaike information criterion; p_starve_ starvation probability; BIC, Bayesian information criterion
