# Supplementary material for: Maintaining Homeostasis by Decision-Making
Source: PLoS Comput Biol. 2015 May 29;11(5):e1004301. doi: 10.1371/journal.pcbi.1004301 (PMC4449003; doi:10.1371/journal.pcbi.1004301)
Supplement: S3 Table — (DOCX) [file pcbi.1004301.s006.docx]

**S3 Table.** Model comparison for all data points: individual model fits according to BIC

|  | BIC (smaller is better) | | | | | | | | |
| --- | --- | --- | --- | --- | --- | --- | --- | --- | --- |
|  | Family 1 | | | | Family 2 | | Family 3 | | |
|  | Moments without p_starve_ | | | | Rank-dependent utility | | Moments and p_starve_ | | |
|  | Model | Model | Model | Model | Model | Model | Model | Model | Model |
|  | 1 | 2 | 3 | 4 | 5 | 6 | 7 | 8 | 9 |
|  | EV | EV | EV | EV | Prelec-I | Prelec-II | EV | EV | EV |
|  |  | Var | Skw | Var |  |  | p_starve_ | Var | Var |
|  |  |  |  | Skw |  |  |  | p_starve_ | Skw |
|  |  |  |  |  |  |  |  |  | p_starve_ |
| P1 | 615 | 614 | **611** | 614 | 617 | 618 | 611 | 614 | 617 |
| P2 | 560 | 562 | 562 | 565 | 561 | 564 | **560** | 564 | 565 |
| P3 | 620 | 593 | 565 | 568 | 572 | 576 | **561** | 562 | 566 |
| P4 | 637 | 572 | 551 | 549 | 523 | 525 | 517 | 519 | **505** |
| P5 | 468 | 461 | 454 | 457 | 454 | 457 | **451** | 454 | 457 |
| P6 | 631 | 574 | 525 | 528 | 531 | 530 | **523** | 525 | 527 |
| P7 | 667 | 600 | 612 | 597 | 598 | 601 | 599 | **595** | 598 |
| P8 | 659 | 547 | 491 | 489 | 494 | 497 | **489** | 492 | 492 |
| P9 | 657 | 417 | 318 | 300 | 284 | **283** | 286 | 288 | 291 |
| P10 | **664** | 665 | 664 | 668 | 667 | 670 | 664 | 668 | 671 |
| P11 | 589 | 575 | 577 | 577 | 580 | 581 | **575** | 576 | 580 |
| P12 | 667 | 597 | 607 | 592 | 592 | 595 | 593 | **589** | 592 |
| P13 | 630 | 410 | 382 | 353 | 328 | 331 | 330 | 327 | **323** |
| P14 | 659 | 540 | 481 | 479 | 467 | 470 | **465** | 468 | 472 |
| P15 | 425 | 427 | 421 | 421 | 423 | **415** | 425 | 427 | 420 |
| P16 | 667 | 620 | **564** | 568 | 573 | 567 | 569 | 571 | 570 |
| P17 | 635 | 578 | 476 | **476** | 505 | 491 | 490 | 482 | 477 |
| P18 | 650 | 556 | 500 | 501 | **466** | 469 | 483 | 486 | 523 |
| P19 | 650 | 609 | 599 | 598 | 568 | **566** | 576 | 580 | 571 |
| P20 | 649 | 595 | 537 | 540 | **532** | 534 | 532 | 532 | 535 |
| P21 | 662 | 610 | 577 | 579 | 564 | **562** | 566 | 568 | 572 |
| P22 | **460** | 462 | 461 | 465 | 465 | 468 | 461 | 465 | 468 |

Smaller BIC values indicate more evidence for the respective model. The BIC values of the winning models according to fixed-effects analyses are written in bold font. The models included free parameters for the respective variables listed. BIC, Bayesian information criterion; EV, expected value; Var, variance; Skw, skewness; p_starve_ starvation probability
