# Supplementary material for: Maintaining Homeostasis by Decision-Making
Source: PLoS Comput Biol. 2015 May 29;11(5):e1004301. doi: 10.1371/journal.pcbi.1004301 (PMC4449003; doi:10.1371/journal.pcbi.1004301)
Supplement: S4 Table — (DOCX) [file pcbi.1004301.s007.docx]

**S4 Table.** Comparison within the winning model family: exceedance probabilities based on AIC

|  | Exceedance probabilities based on AIC (higher is better) | | |
| --- | --- | --- | --- |
|  | Family 3 | | |
|  | Moments and p_starve_ | | |
|  | Model 7 | Model 8 | Model 9 |
|  | EV | EV | EV |
|  | p_starve_ | Var | Var |
|  |  | p_starve_ | Skw |
|  |  |  | p_starve_ |
| All | 0.0147 | 0.0616 | **0.9237** |
| Foraging | 0.0001 | 0.0022 | **0.9977** |
| Casino | 0.2521 | 0.6588 | **0.0891** |
| Foraging-block 1 | 0.0826 | 0.0044 | **0.9130** |
| Foraging-block 2 | 0.0007 | 0.0001 | **0.9992** |
| Casino-block 1 | **0.9515** | 0.0170 | 0.0315 |
| Casino-block 2 | **0.8416** | 0.1422 | 0.0162 |

The highest exceedance probabilities according to random-effects analyses are written in bold font. See Table 4 for results based on BIC. AIC, Akaike information criterion; EV, expected value; Var, variance; Skw, skewness; p_starve_ starvation probability; BIC, Bayesian information criterion
