# Supplementary material for: Maintaining Homeostasis by Decision-Making
Source: PLoS Comput Biol. 2015 May 29;11(5):e1004301. doi: 10.1371/journal.pcbi.1004301 (PMC4449003; doi:10.1371/journal.pcbi.1004301)
Supplement: S5 Table — (DOCX) [file pcbi.1004301.s008.docx]

**S5 Table.** Comparison of an additional model including frame-specific parameters: relative log-group Bayes factors and exceedance probabilities based on AIC

|  | Family 3 | | | Additional model |
| --- | --- | --- | --- | --- |
|  | Moments and p_starve_ | | |  |
|  | Model 7 | Model 8 | Model 9 | Model 10 |
|  | EV | EV | EV | EV |
|  | p_starve_ | Var | Var | forage-p_starve_ |
|  |  | p_starve_ | Skw | casino-p_starve_ |
|  |  |  | p_starve_ |  |
| Relative log-group Bayes factors – all data (smaller is better) | 0 | -30 | -43 | **-850** |
| Exceedance probabilities – all data (higher is better) | 0.0000 | 0.0001 | 0.0076 | **0.9923** |

Log-group Bayes factors based on AIC were calculated relative to the simplest model (Model 7). Smaller log-group Bayes factors indicate more evidence for the respective model versus the baseline model. The log-group Bayes factor of the winning model according to fixed-effects analysis and the highest exceedance probability according to random-effects analysis are written in bold font. See Table 5 for results based on BIC. AIC, Akaike information criterion; EV, expected value; Var, variance; Skw, skewness; p_starve_ starvation probability; BIC, Bayesian information criterion
