# Supplementary material for: Maintaining Homeostasis by Decision-Making
Source: PLoS Comput Biol. 2015 May 29;11(5):e1004301. doi: 10.1371/journal.pcbi.1004301 (PMC4449003; doi:10.1371/journal.pcbi.1004301)
Supplement: S6 Table — (DOCX) [file pcbi.1004301.s009.docx]

**S6 Table.** Comparison of a model based solely on EV with a model based solely on p_starve_

|  | Model 1 | | Model 11 | |
| --- | --- | --- | --- | --- |
|  | EV | | p_starve_ | |
|  | Log-group Bayes factors (smaller is better) | Exceedance probabilities (higher is better) | Log-group Bayes factors (smaller is better) | Exceedance probabilities (higher is better) |
| All | 0 | 0.1996 | **-490** | **0.8004** |
| Foraging | 0 | 0.0177 | **-812** | **0.9823** |
| Casino | **0** | **0.8237** | 161 | 0.1763 |
| Foraging-block 1 | 0 | 0.0596 | **-407** | **0.9404** |
| Foraging-block 2 | 0 | 0.0630 | **-448** | **0.9370** |
| Casino-block 1 | **0** | **0.6034** | 44 | 0.3966 |
| Casino-block 2 | **0** | **0.9249** | 106 | 0.0751 |

Log-group Bayes factors based on BIC were calculated relative to the simpler model (Model 1). Smaller log-group Bayes factors indicate more evidence for the respective model versus the baseline model. The log-group Bayes factor of the winning model according to fixed-effects analysis and the higher exceedance probability according to random-effects analysis are written in bold font. BIC, Bayesian information criterion; EV, expected value; p_starve_ starvation probability
