# Supplementary material for: Maintaining Homeostasis by Decision-Making
Source: PLoS Comput Biol. 2015 May 29;11(5):e1004301. doi: 10.1371/journal.pcbi.1004301 (PMC4449003; doi:10.1371/journal.pcbi.1004301)
Supplement: S7 Table — (DOCX) [file pcbi.1004301.s010.docx]

**S7 Table.** Comparison of a model with day-specific weighting parameters for p_starve_

|  |  | | Additional model with day-specific weighting parameters | |
| --- | --- | --- | --- | --- |
|  | Model 7 | | Model 12 | |
|  | EV | | EV | |
|  | p_starve_ | | one-day-p_starve_ | |
|  |  | | two-days-p_starve_ | |
|  |  | | three-days-p_starve_ | |
|  | Log-group Bayes factors  (smaller is better) | Exceedance probabilities (higher is better) | Log-group Bayes factors (smaller is better) | Exceedance probabilities (higher is better) |
| All | **0** | **0.9902** | 248 | 0.0098 |
| Foraging | **0** | **0.9474** | 18 | 0.0526 |
| Casino | **0** | **0.9987** | 159 | 0.0013 |
| Foraging-block 1 | **0** | **0.9966** | 74 | 0.0034 |
| Foraging-block 2 | 0 | **0.8719** | **-11** | 0.1281 |
| Casino-block 1 | **0** | **1.0000** | 87 | 0.0000 |
| Casino-block 2 | **0** | **1.0000** | 72 | 0.0000 |

Log-group Bayes factors based on BIC were calculated relative to the simpler model (Model 7). Smaller log-group Bayes factors indicate more evidence for the respective model versus the baseline model. The log-group Bayes factor of the winning model according to fixed-effects analysis and the higher exceedance probability according to random-effects analysis are written in bold font. BIC, Bayesian information criterion; EV, expected value; p_starve_ starvation probability
