# Supplementary material for: Maintaining Homeostasis by Decision-Making
Source: PLoS Comput Biol. 2015 May 29;11(5):e1004301. doi: 10.1371/journal.pcbi.1004301 (PMC4449003; doi:10.1371/journal.pcbi.1004301)
Supplement: S8 Table — (DOCX) [file pcbi.1004301.s011.docx]

**S8 Table.** Comparison of models based on the assumption that participants falsely included values below zero in the outcome distributions in the foraging frame.

|  | Family 1 | | | | Family 3 | | |
| --- | --- | --- | --- | --- | --- | --- | --- |
|  | Moments without p_starve_ | | | | Moments and p_starve_ | | |
|  | Model | Model | Model | Model | Model | Model | Model |
|  | 1 | 2 | 3 | 4 | 7 | 8 | 9 |
|  | EV | EV | EV | EV | EV | EV | EV |
|  |  | Var | Skw | Var | p_starve_ | Var | Var |
|  |  |  |  | Skw |  | p_starve_ | Skw |
|  |  |  |  |  |  |  | p_starve_ |
| Model family comparison: relative log-group Bayes factors (smaller is better) | | | | | | | |
| Outcome distributions *without* values < 0 | 0 | -884 | -1390 | -1437 | -1511 | -1532 | **-1569** |
| Outcome distributions *including* values < 0 | 0 | -945 | -1366 | -1465 | -1514 | -1562 | **-1610** |
| Model family comparison: exceedance probabilities (higher is better) | | | | | | | |
| Outcome distributions *without* values < 0 | 0.0071 | | | | **0.9929** | | |
| Outcome distributions *including* values < 0 | 0.0016 | | | | **0.9984** | | |
| Comparison within the winning model family: exceedance probabilities (higher is better) | | | | | | | |
| Outcome distributions *without* values < 0 | - | | | | **0.8627** | 0.0108 | 0.1265 |
| Outcome distributions *including* values < 0 | - | | | | **0.7029** | 0.0820 | 0.2151 |

Please note that only data from the foraging frame was included since participants had no reason at all to falsely include values below zero in the casino frame. Log-group Bayes factors based on BIC were calculated relative to the simplest model (Model 1). The difference in the log-group Bayes factor of Model 1 based on outcome distributions *without* values < 0 versus that of Model 1 based on outcome distributions *including* values < 0 was +7. The data for the models based on outcome distributions *without* values < 0 are partly presented in Tables 2 and 4 and are included here for comparison. Smaller log-group Bayes factors indicate more evidence for the respective model versus the baseline model. The log-group Bayes factor of the winning model according to fixed-effects analysis and the highest exceedance probability according to random-effects analysis are written in bold font. BIC, Bayesian information criterion; EV, expected value; Var, variance; Skw, skewness; p_starve_ starvation probability
